# Earth's Impact Events Through Geologic Time: A List of Recommended Ages for Terrestrial Impact Structures and Deposits

**DOI:** 10.1089/ast.2019.2085

**Published:** 2020-01-20

**Authors:** Martin Schmieder, David A. Kring

**Affiliations:** ^1^ Lunar and Planetary Institute—USRA, Houston, Texas.; ^2^ NASA Solar System Exploration Research Virtual Institute (SSERVI).

**Keywords:** Impact craters, Ejecta, Ages, Geochronology, Terrestrial, Cratering record

## Abstract

This article presents a current (as of September 2019) list of recommended ages for proven terrestrial impact structures (*n* = 200) and deposits (*n* = 46) sourced from the primary literature. High-precision impact ages can be used to (1) reconstruct and quantify the impact flux in the inner Solar System and, in particular, the Earth–Moon system, thereby placing constraints on the delivery of extraterrestrial mass accreted on Earth through geologic time; (2) utilize impact ejecta as event markers in the stratigraphic record and to refine bio- and magneto-stratigraphy; (3) test models and hypotheses of synchronous double or multiple impact events in the terrestrial record; (4) assess the potential link between large impacts, mass extinctions, and diversification events in the biosphere; and (5) constrain the duration of melt sheet crystallization in large impact basins and the lifetime of hydrothermal systems in cooling impact craters, which may have served as habitats for microbial life on the early Earth and, possibly, Mars.

## 1. Introduction

**FIG. 1.**
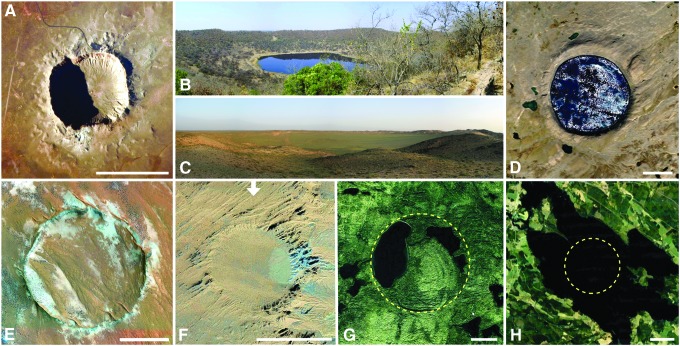
Degradation of terrestrial impact craters over time, exemplified by a number of simple, bowl-shaped impact craters that are most easily erased from the terrestrial impact cratering record. The same principle applies to complex impact craters on Earth larger than ∼2 to 4 km in diameter (not shown here). **(A)** The ∼1.2 km-diameter and roughly 50 kyr-old Meteor Crater (*aka* Barringer Meteorite Crater) in Arizona is one of the best-preserved simple impact craters on Earth (*e.g*., Shoemaker, 1960; Kring, 2017b). Its ejecta blanket forms a hummocky terrain surrounding the crater. Note the pronounced topography of the crater indicated by low-angle sunlight coming from the WSW. ISS spacecraft image ISS-038-E-67508. **(B)** The 1.13 km-diameter and ∼220 kyr-old Tswaing impact crater in South Africa (*e.g*., Brandt and Reimold, 1995), with its crater bowl seen from the uplifted crater rim. After more than 2000 centuries of erosion, its topographic features have been smoothed out considerably compared with Meteor Crater. Photo taken during 2008 field expedition. **(C)** The ∼1.3 km-diameter Tavan Khar Ovoo (*aka* Tabun Khara Obo) impact crater in Mongolia (Masaitis, 1999; Schmieder *et al.*, 2013). The crater rim is less pronounced than those at Meteor Crater and Tswaing, and the crater bowl is filled with a thick pile of postimpact sediments, mainly lake sediments and alluvium. The age of the crater is somewhat uncertain but likely on the order of a few Myr. Photo taken during 2011 field expedition. **(D)** Satellite image of the ∼3.4 km-diameter and ∼1.1 Myr-old New Québec (Pingualuit) impact crater in Canada (*e.g*., Grieve *et al.*, 1991; Marvin and Kring, 1992; Grieve, 2006), with parts of its elevated crater rim preserved despite Pleistocene glacial overprint. The crater is filled with a modern-day lake. **(E)** The ∼2.5 km-diameter and ∼4 to 5 Myr-old Roter Kamm impact crater in Namibia (*e.g*., Grant *et al.*, 1997; Miller, 2010). After a few million years of surface exposure, the crater has been modified by notable degradation and postimpact sediment infill. **(F)** Satellite view of the Tavan Khar Ovoo crater shown in **(C)**, characterized by a level of erosion similar to that of the New Québec crater and the Roter Kamm crater. **(G)** Satellite image of the 3.8 km-diameter and ∼453 Ma Brent crater in Canada, filled with postimpact sediments and today partly occupied by lakes. After several hundred Myr, this crater (dashed circle) is vaguely recognizable by its morphology, and most of the impact crater geology is known from drillings (Grieve, 1978, 2006). **(H)** Satellite image of the recently discovered ∼2.6 km-diameter Summanen impact structure in Finland of uncertain age (Plado *et al.*, 2018). After perhaps hundreds of millions of years of exposure, the crater (dashed circle) has been significantly overprinted by erosion, sedimentary infill, and glaciation and is today concealed by a lake. The discovery and characterization of such old, “invisible” small impact structures usually requires detailed geologic mapping and field work, as well as drilling. Scale bars are 1 km.

Impact cratering is a fundamental process in the Solar System, shaping asteroids, planets, and their satellites (*e.g*., Baldwin, 1971; Shoemaker, 1983; Melosh, 1989; Ryder, 1990; French, 1998, 2004; Canup and Asphaug, 2001; Kring and Cohen, 2002; Osinski and Pierazzo, 2012). Unlike the Moon, whose surface has been modified by numerous large and small impacts for more than 4 billion years (Ga, Gyr) (*e.g*., Stöffler *et al.*, 2006), the Earth has retained a limited impact cratering record due to tectonic recycling of the crust, erosion, and the burial of impact craters underneath layers of sediment and lava (*e.g*., Grieve, 1987, 2001a, 2001b) (Fig. 1).

**FIG. 2.**
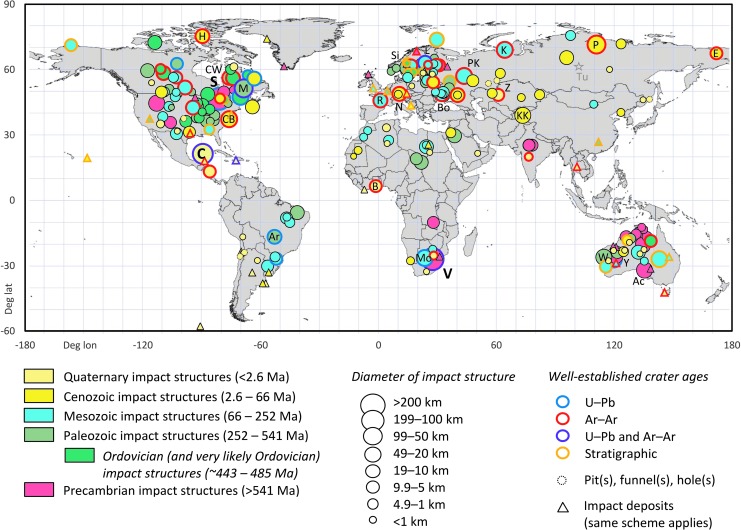
Map of impact structures (*n* = 200) and deposits (*n* = 46) on Earth (including prominent impact holes, funnels, and pits) and their best-estimate ages. For poorly constrained ages, the stratigraphic maximum age was chosen. Only a few representative ejecta localities are shown (*e.g*., Thailand for the Australasian tektite strewn field) because some distal ejecta deposits, such as the end-Cretaceous Chicxulub ejecta (plotted at Beloc, Haiti; yellow-green symbols) or the Popigai-derived Upper Eocene clinopyroxene spherules (plotted near Hawaii), have a global or semiglobal distribution. Some prominent terrestrial impact structures are labeled as follows: Ac, Acraman; Ar, Araguainha; B, Bosumtwi; Bo, Boltysh; C, Chicxulub; CB, Chesapeake; CW, West and East Clearwater Lake; E, El′gygytgyn; H, Haughton; K, Kara; KK, Kara-Kul; L, Lappajärvi; M, Manicouagan; Mo, Morokweng; N, Nördlinger Ries; P, Popigai; PK, Puchezh-Katunki; R, Rochechouart; S, Sudbury; Si, Siljan; V, Vredefort; W, Woodleigh; Y, Yarrabubba; Z, Zhamanshin. The gray star symbol marks the site of the June 30, 1908, Tunguska (Tu) explosion that downed trees in a vast area but left no impact structure on the ground. Compare Table 1 with ages for impact structures and Table 2 with ages for impact deposits.

Before ∼3.7 Ga before present, when most of the large lunar impact basins were created, impact rates in the Earth–Moon system were much higher than they are today (*e.g*., Turner *et al.*, 1973; Tera *et al.*, 1974; Ryder, 1990; Kring and Cohen, 2002; Grieve *et al.*, 2006; Johnson and Melosh, 2012; Bottke and Norman, 2017). However, no traces of those Hadean (>4.0 Ga) and Eoarchean (4.0–3.6 Ga) impacts on the early Earth are currently known in the geologic record (*e.g*., Koeberl, 2006). Only 200 proven impact structures (counting fields of small impact craters produced during the same event as one) and 46 individual horizons of proximal and distal impact ejecta (again, counting layers with the same age at different localities as one) have thus far been recognized on our planet (Fig. 2). Those impact structures and deposits span a time from more than ∼3.4 Ga, represented by Paleoarchean impact spherule layers in South Africa and Western Australia produced by large impacts (*e.g*., Glass and Simonson, 2012, 2013), to roughly 6 years ago when the Chelyabinsk airburst in Russia (February 15, 2013) shattered windows and its main stony meteorite mass produced an ∼7 m-wide circular impact penetration hole in frozen Lake Chebarkul (*e.g*., Borovička *et al.*, 2013; Popova *et al.*, 2013).

**Table 1.** List of Proven Terrestrial Impact Structures, Select Age Constraints, and Recommended Impact Ages Sorted by Age

No	Impact structure	Country	Latitude	Longitude	Diameter (km)	Type of target rock^a^	Type of impactor^b^	Stratigraphic age constraints	Radioisotopic age constraints	Other age constraints	Recommended age (Ma)	Recommended age reference^c^	Pre-recalculation age (Ma)
1	Chelyabinsk^d^	Russia	4°58′N	60°18′E	0.007	Ice	LL-chondrite	Recent		Fall February 15, 2013, main mass left 8 m-wide temporary hole in frozen Lake Chebarkul	0.000006	Borovička *et al.* (2013)	
2	Carancas	Peru	16°40′S	69°02′W	0.0135	Sedimentary	H-chondrite	Recent		Fall September 15, 2007	0.000012	Tancredi *et al.* (2009)	
3	Sterlitamak^e^	Russia	53°40′N	55°59′E	0.0094	Sedimentary (soil, loam)	IIIAB iron	Recent		Fall May 17, 1990	0.000029	Petaev (1992)	
4	Sikhote Alin (Field)^f^	Russia	46°09′N	134°39′E	0.027	Crystalline	IIAB iron	Recent		Fall February12, 1947	0.000072	Krinov (1971)	
5	Imilac^e^	Chile	24°12′S	68°48′W	0.015	Crystalline (volcanic, soil)	Pallasite	Recent		Found 1822 AD; fall produced ∼15 m-wide impact pit, ∼4 m deep	>0.0002	Buchwald (1973); Bevan (2006)	
6	Sobolev^e^	Russia	46°17′N	137°54′E	0.053	Sedimentary	Iron?	Recent		Trees in crater	0.00030–0.00025	Yarmolyuk (1951); Khryanina (1981)	
7	Wabar (Field)^f^	Saudi Arabia	21°30′N	50°28′E	0.116	Sedimentary (sand)	IIIAB iron	Historical?		Luminescence dating, historical	∼0.0003	Basurah (2003); Prescott *et al.* (2004)	
8	Whitecourt	Canada	54°00′N	115°36′W	0.036	Sedimentary	IIIAB iron		^14^C (charcoal)		<0.0011	Herd *et al.* (2008)	
9	Dalgaranga	Australia	27°45′S	117°05′E	0.021	Crystalline	Mesosiderite	Quaternary		Preservation of morphology	<0.003?	Shoemaker and Shoemaker (1988)	
10	Kamil	Egypt	22°01′N	26°05′E	0.045	Sedimentary	Iron (ataxite)			Thermoluminescence dating	≤0.004	Sighinolfi *et al.* (2015)	
11	Kaali (Kaalijärv) (Field)^f^	Estonia	58°22′N	22°40′E	0.11	Sedimentary	IAB iron		^14^C (charcoal)		∼0.00324	Losiak *et al.* (2016)	
12	Vaca Muerta (Field)^e^	Chile	25°45′S	70°30′W	0.007	Crystalline (volcanic)	Mesosiderite	Quaternary?	^14^C	Fall produces field of pits, largest is ∼7.16 m in diameter and 1.35 m deep	∼0.0035	Pedersen *et al.* (1992)	
13	Campo del Cielo (Field)^f^	Argentina	27°38′S	61°42′W	0.115	Sedimentary	IAB iron	Quaternary	^14^C (charcoal)		∼0.004	Romaña and Cassidy (1973)	
14	Veevers	Australia	22°58′S	125°22′E	0.08	Sedimentary	IIAB iron			Preservation of ejecta blanket	∼0.004?	Shoemaker and Shoemaker (1988); Haines (2005); Shoemaker *et al.* (2005)	
15	Morasko (Field)^f^	Poland	52°29′N	27°24′E	0.1	Sedimentary	IAB iron	Quaternary		Luminescence dating	∼0.005	Stankowski *et al.* (2007)	
16	Ilumetsa (Field)	Estonia	57°57′N	16°54′E	0.08	Sedimentary	Unknown	Quaternary	^14^C (charcoal)		∼0.007	Raukas *et al.* (2001); Losiak *et al.* (2017); A. Losiak (2019), personal communication	
17	Macha (Field)^f^	Russia	60°05′N	117°39′E	0.3	Sedimentary	Iron		^14^C (charcoal)		0.007315 ± 0.00008	Gurov *et al.* (1987); Gurov and Gurova (1998)	
18	Haviland^e^	United States	37°35′N	99°10′W	0.015	Sedimentary	Pallasite (Brenham)			Cosmogenic nuclides (^14^C)	0.020 ± 0.002	Honda *et al.* (2002)	
19	Boxhole	Australia	22°37′S	135°12′E	0.185	Crystalline	IIIAB iron			^10^Be/^26^Al exposure age	0.030 ± 0.005	Shoemaker *et al.* (1990)	
20	Henbury (Field)^f^	Australia	24°35′S	133°09′E	0.157	Sedimentary	IIIAB iron			Cosmogenic nuclides (^14^C)	0.042 ± 0.019	Kohman and Goel (1963)	
21	Amguid	Algeria	26°05′N	4°23′E	0.45	Sedimentary		Lower Devonian target rocks		Freshness of crater morphology	≤0.1?	Lambert *et al.* (1980)	
22	Hickman	Australia	23°02′S	119°41′E	0.26	Mixed (banded iron formation, rhyolite)	Iron?	Paleoproterozoic (Boolgeeda Iron Fm.) or younger		Plumbing of local drainage system	≤0.1?	Glikson *et al.* (2008); Haines (2014)	
23	Barringer (Meteor Crater)	United States	35°02′N	111°01′W	1.186	Sedimentary	IAB iron	Early Triassic to Quaternary		^36^Cl surface exposure; ^10^Be, ^26^Al exposure	∼0.056?; 0.0611 ± 0.0048	Sutton (1985); Marrero *et al.* (2010); Kring (2017b) and references therein; Barrows *et al.* (2019)	
24	Odessa (field)^f^	United States	31°45′N	102°29′W	0.168	Sedimentary	IAB iron			Optically stimulated luminescence	0.0635 ± 0.0045	Holliday *et al.* (2005)	
25	Wolfe Creek (Kandimalal)	Australia	19°18′S	127°46′E	0.875	Sedimentary	IIIAB iron			Optically stimulated luminescence, ^10^Be, ^26^Al exposure	0.120 ± 0.009	Shoemaker *et al.* (1990, 2005); Barrows *et al.* (2019)	
26	Tswaing (Pretoria Saltpan)	South Africa	25°24′S	28°05′E	1.13	Crystalline	Chondrite			Glass fission track	0.220 ± 0.104	Storzer *et al.* (1999); Jourdan *et al.* (2007)	
27	Kalkkop	South Africa	32°43′S	24°26′E	0.64	Sedimentary	Chondrite?		U–Th series dating of limestone		0.25 ± 0.05	Reimold *et al.* (1998)	
28	Lonar	India	19°59′N	76°31′E	1.83	Crystalline (basalt)	Carbonaceous chondrite?		Ar–Ar (impact melt rock)		0.576 ± 0.047	Jourdan *et al.* (2011), recalculated	0.570 ± 0.047
29	Monturaqui	Chile	23°56′S	68°17′W	0.46	Crystalline (granite, volcanics)	IAB? iron		(U–Th)/He (zircon and apatite from impactite)		0.663 ± 0.09	Ukstins Peate *et al.* (2010)	
30	Pantasma	Nicaragua	13°12′N	85°57′W	14	Crystalline (volcanic)			Ar-Ar (impact glass)		0.815 ± 0.011	Rochette *et al.* (2019)	
31	Zhamanshin	Kazakhstan	48°24′N	60°58′E	14	Mixed	Carbonaceous chondrite		Ar-Ar (impact glass)		0.91 ± 0.14	Deino *et al.* (1990), recalculated	0.87 ± 0.13 (range)
32	Bosumtwi	Ghana	6°32′N	1°25′W	10.5	Crystalline	Chondrite? Iron?		Ar-Ar (Ivory Coast tektites)		1.13 ± 0.10	Jourdan (2012) after Koeberl *et al.* (1997b)	
33	New Québec (Pingualuit)	Canada	61°17′N	73°40′W	3.44	Crystalline	Chondrite (L?)		Ar–Ar (impact melt rock)		1.4 ± 0.1	Grieve *et al.* (1991)	
34	Talemzane (Maâdna)	Algeria	33°19′N	4°02′E	1.75	Sedimentary		Eocene or younger		Preservation of crater morphology	≤3?	Lambert *et al.* (1980); Reimold and Koeberl (2014)	
35	Tenoumer	Mauritania	22°55′N	10°24′W	1.9	Crystalline			Ar-Ar (impact melt rock)		1.57 ± 0.14	Schultze *et al.* (2016)	
36	Aouelloul	Mauritania	20°15′N	12°41′W	0.36	Sedimentary	Iron		K-Ar (impact glass)		3.1 ± 0.3	Fudali and Cressy (1976)	
37	El'gygytgyn	Russia	67°30′N	172°05′E	18	Crystalline (volcanic)	Achondrite?		Ar-Ar (impact melt rock)		3.65 ± 0.08	Layer (2000)	3.58 ± 0.04
38	Roter Kamm	Namibia	S27°46′	16°18′E	2.5	Mixed			Ar-Ar (impact melt rock)		3.8 ± 0.3; ∼4 to 5	Koeberl *et al.* (1993); Hecht *et al.* (2008)	3.7 ± 0.3
39	Bigach	Kazakhstan	48°30′N	82°00′E	7	Mixed		Miocene or younger			5 ± 3	Masaitis (1999)	
40	Karla	Russia	54°54′N	48°00′E	12	Sedimentary		Miocene to Pliocene			5 ± 1	Masaitis (1999)	
41	Xiuyan	China	40°21′N	123°27′E	1.8	Crystalline		Proterozoic to Quaternary	^14^C (charcoal)		5–0.05?	Chen *et al.* (2011); Liu *et al.* (2013)	
42	Shunak	Kazakhstan	47°12′N	72°42′E	2.8	Crystalline (volcanic)		Middle/Late Devonian to Miocene		Crater morphology	12 ± 5	Masaitis *et al.* (1980); Masaitis (1999)	
43	Nördlinger Ries (Ries crater)	Germany	48°53′N	10°37′E	24	Mixed	No contamination? (achondrite?)	Middle Miocene	Ar-Ar (moldavite tektites)		14.808 ± 0.038	Schmieder *et al.* (2018a, 2018b)	
44	Steinheimer Becken (Steinheim Basin)	Germany	48°40′N	10°04′E	3.8	Sedimentary	Pallasite?	Miocene crater lake sediments		Presumably synchronous with Ries impact	14.808 ± 0.038?	Stöffler *et al.* (2002); Schmieder *et al.* (2018a, 2018b)	
45	Haughton	Canada	75°22′N	89°41′W	24	Mixed	No contamination		Ar-Ar (impactites)		23.4 ± 1.0	Jessberger (1988); Young *et al.* (2013)	23.4 ± 1.0
46	Jebel Waqf as Suwwan	Jordan	31°03′N	36°48′E	6	Sedimentary		Middle Eocene or younger			<48	Salameh *et al.* (2008)	
47	Karakul (Kara-Kul)	Tajikistan	39°01′N	73°27′E	52	Crystalline				Tectonic history	<60	Gurov *et al.* (1993); Baratoux *et al.* (2012)	
48	Logoisk	Belarus	54°12′N	27°48′E	17	Mixed			Ar-Ar (impact glass)		30.0 ± 0.5	Jourdan *et al.* (2012) after Sherlock *et al.* (2009)	
49	Beyenchime-Salaatin	Russia	71°50′N	123°30′E	8	Sedimentary				Cenozoic? (preservation of crater)	33 ± 33	Masaitis (1999)	
50	Eagle Butte	Canada	49°42′N	110°35′W	10	Sedimentary		Late Cretaceous or younger			<65	Grieve (2006)	
51	Gusev	Russia	N 48°21′	E 40°14′	3	Sedimentary		Younger than Late Cretaceous			<66	Movshovic *et al.* (1991)	
52	Chesapeake (Chesapeake Bay)	United States	37°15′N	76°05′W	∼40 to 45	Sedimentary	Chondrite (L?)		Ar-Ar (tektites and impact melt rock)		34.86 ± 0.32	Assis Fernandes *et al.* (2019)	
53	Chukcha (Chykcha)	Russia	75°42′N	97°48′E	6	Mixed		Cretaceous or younger			<70	Vishnevsky (1995)	
54	Maple Creek	Canada	49°48′N	109°06′W	6	Sedimentary		Maastrichtian or younger			<72	Grieve (2006)	
55	Popigai	Russia	71°30′N	111°00′E	100	Mixed	Chondrite (H or L?)		Ar-Ar (impact melt rock)		36.63 ± 0.92	Bottomley *et al.* (1997), recalculated, mean of 4 plateau ages	35.7 ± 0.2
56	Wanapitei	Canada	46°45′N	80°45′W	7.5	Crystalline	Chondrite (L or LL?)		Ar-Ar (impact melt rock)		37.7 ± 1.2	Grieve (2006) after Bottomley *et al.* (1979), recalculated	37.2 ± 1.2
57	Mistastin	Canada	55°53′N	63°18′W	28	Crystalline	No contamination? Achondrite? Iron?		U–Pb (CA-TIMS, melt rock zircon)		37.83 ± 0.05	Sylvester *et al.* (2013)	
58	Connolly Basin	Australia	23°32′S	124°45′E	9	Sedimentary		Paleogene			∼66 to 23	Shoemaker and Shoemaker (1986)	
59	Logancha	Russia	65°30′N	95°48′E	20	Mixed		Paleogene			∼66 to 23	Masaitis (1999)	
60	Tin Bider (Tademaït)	Algeria	27°36′N	5°07′E	6	Sedimentary		Coniacian or younger			≤90	Lambert *et al.* (1981)	
61	Chiyli (Shyili)	Kazakhstan	49°10′N	57°51′E	5.5	Sedimentary		Early to Middle Eocene			∼56 to 41	Vishnevsky (2007)	
62	Santa Marta	Brazil	10°10′S	45°14′W	10	Sedimentary		Late Cretaceous (Posse Fm.) to Neogene (Chapadão Fm.)			<100	Oliveira *et al.* (2017); Crósta *et al.* (2019b)	
63	Kamensk	Russia	48°20′N	40°15′E	25	Sedimentary			Ar-Ar (impact glass)		50.37 ± 0.40	Jourdan *et al.* (2012) after Izett *et al.* (1994)	
64	Montagnais	Canada	42°53′N	64°13′W	45	Sedimentary (shelf)			Ar-Ar (impact melt rock)		51.1 ± 1.6	Bottomley and York (1988), recalculated	50.5 ± 1.6
65	Goat Paddock	Australia	18°20′S	126°40′E	5.1	Sedimentary		Early Eocene (palynology)			∼56 to 48	Milton and Macdonald (2005)	
66	Ragozinka	Russia	58°18′N	62°00′E	9	Mixed		Thanetian, Early Eocene (Serov Suite)			∼59 to 56	Vishnevsky (1999)	
67	Sierra Madera	United States	30°36′N	102°55′W	13	Sedimentary		Albian (Georgetown Fm.) or younger			<113	Wilshire *et al.* (1972)	
68	Marquez	United States	31°17′N	96°18′W	13	Sedimentary		Around Paleocene/Eocene transition		Apatite fission track	58.3 ± 3.1	Sharpton and Gibson (1990); McHone and Sorkhabi (1994)	
69	BP structure	Libya	25°19′N	24°20′E	2	Sedimentary		Early Cretaceous or younger (Nubian Sandstone)			≤120	Koeberl *et al.* (2005b)	
70	Oasis	Libya	24°35′N	24°24′E	18	Sedimentary		Early Cretaceous or younger (Nubian Sandstone)			≤120	Koeberl *et al.* (2005b)	
71	Mount Toondina	Australia	27°57′S	135°22′E	4	Sedimentary		Aptian to Albian, Early Cretaceous, or younger (Bulldog Shale)			<125	Plescia *et al.* (1994)	
72	Chicxulub	Mexico	21°20′N	89°30′W	180	Mixed	Carbonaceous chondrite?	K/T (K/Pg) boundary	Ar-Ar (impact melt rock and glassy microtektites)		66.052 ± 0.043	Swisher *et al.* (1992), recalculated Renne *et al.* (2013, 2018); Sprain *et al.* (2015, 2018); Clyde *et al.* (2016)	
73	Boltysh	Ukraine	48°45′N	32°10′E	24	Crystalline	Chondrite?	Slightly older than Chicxulub (distal K/T ejecta in post-impact sediments)	Ar-Ar (impact melt rock)		65.80 ± 0.67	Kelley and Gurov (2002), recalculated Jourdan (2012)	
74	Cerro do Jarau	Brazil	30°12′S	56°32′W	13.5	Mixed		Early Cretaceous (Serra Geral Fm.) or younger			≤135	Crósta *et al.* (2019a)	
75	Kara	Russia	69°05′N	64°18′E	65	Mixed	Chondrite?		Ar-Ar (impact melt rock)		70.7 ± 2.2	Trieloff *et al.* (1998), recalculated	70.3 ± 2.2
76	Manson	United States	42°35′N	94°31′W	35	Mixed	Chondrite		Ar-Ar (sanidine in melt breccia)		75.9 ± 0.1	Izett *et al.* (1998), recalculated	74.1 ± 0.1
77	Lappajärvi	Finland	63°09′N	23°42′E	23	Mixed	H-chondrite		U-Pb (zircon in impact melt rock)		77.85 ± 0.78	Schmieder and Jourdan (2013a); Kenny *et al.* (2019b)	
78	Zeleny Gai	Ukraine	48°42′N	32°54′E	3.5	Crystalline		Archean to Paleogene			80 ± 20?	Masaitis (1999)	
79	Wetumpka	United States	32°31′N	86°11′W	6.5	Mixed	Chondrite?	Close to Santonian/Campanian boundary	(U–Th)/He (apatite and zircon)		∼83.5	King *et al.* (2007); Wartho *et al.* (2012)	
80	Suvasvesi North	Finland	62°39′N	28°10′E	3.5	Crystalline			Ar-Ar (impact melt rock)		∼85	Schwarz *et al.* (2016a); Schmieder *et al.* (2016b)	
81	Yallalie	Australia	30°28′S	115°47′E	15	Sedimentary		Coniacian, Late Cretaceous			89.8–83.6 Ma	Cox *et al.* (2019)	
82	Agoudal	Morocco	31°59′N	5°31′W	1–3	Sedimentary		Middle Jurassic or younger			≤174	Chennaoui Aoudjehane *et al.* (2016)	
83	Kgagodi	Botswana	22°29′S	27°35′E	3.5	Crystalline		Early Jurassic (Karoo dolerite) to Paleogene			≤180	Brandt *et al.* (2002)	
84	Avak	United States	71°15′N	156°36′W	12	Sedimentary		Turonian, Late Cretaceous (palynology)			94–90	Banet and Fenton (2008)	
85	Upheaval Dome	United States	38°26′N	109°54′W	10	Sedimentary		Early Jurassic (Toarcian) or younger			<183	Kriens *et al.* (1999)	
86	Deep Bay	Canada	56°24′N	102°59′W	13	Crystalline		Late Albian to Early Cenomanian (palynology)			102–95	Grieve (2006)	
87	Rotmistrovka	Ukraine	49°00′N	32°00′E	2.7	Crystalline		Early Cretaceous to Turonian			∼145 to 94	Masaitis (1999)	
88	Vista Alegre	Brazil	25°57′S	52°41′W	9.5	Mixed		Early Cretaceous (∼134 Ma Serra Geral Fm.) or younger	Ar-Ar (minimum age)		∼111 to 134	Crósta *et al.* (2019b)	
89	Mien	Sweden	56°25′N	14°52′E	9	Crystalline	Stone?		Ar–Ar (impact melt rock)		122.4 ± 2.3	Bottomley *et al.* (1990), recalculated	121.0 ± 2.3
90	Vargeão	Brazil	26°50′S	52°07′W	12			Early Cretaceous (∼134 Ma Serra Geral Fm.) or younger	U-Pb (zircon in impact melt breccia)		123.0 ± 1.4	Nédélec *et al.* (2013)	
91	Serra da Cangalha	Brazil	8°05′S	46°52′W	12	Sedimentary		Late Permian or younger			≤250	Kenkmann *et al.* (2011)	
92	Tookoonooka	Australia	27°00′S	143°00′E	55	Sedimentary		Barrêmian/Aptian boundary			125 ± 1; ∼121.8 to 123.8?	Bron and Gostin (2012); Olierook *et al.* (2019)	
93	Dellen	Sweden	61°55′N	16°39′E	19	Crystalline	Stone?		Ar-Ar (impact melt rock)		140.82 ± 0.51	Mark *et al.* (2014)	
94	Mjølnir	Norway	73°48′N	29°40′E	20–40	Sedimentary (sea floor)		Early Berriasian (Volgian/Ryazanian boundary)			∼143.0 ± 2	Smelror *et al.* (2001)	
95	Morokweng	South Africa	26°28′S	23°32′E	70	Crystalline	LL-chondrite		U-Pb (CA-ID-TIMS, melt-grown zircon)		146.06 ± 0.16	Hart *et al.* (1997); Koeberl *et al.* (1997a); Kenny *et al.* (2019a)	
96	Des Plaines	United States	42°03′N	87°52′W	8	Sedimentary		Post-Pennsylvanian			<299	Emrich and Bergstrom (1962)	
97	Kentland	United States	40°45′N	87°24′W	13	Sedimentary		Younger than Mississippian, older than Pleistocene			∼300 to 1	Weber *et al.* (2005)	
98	Middlesboro	United States	36°37′N	83°44′W	6	Sedimentary		Younger than Pennsylvanian			<299	Englund and Roen (1963)	
99	Riachão	Brazil	7°43′S	46°39′W	4.5	Sedimentary		Early Permian (Pedra do Fogo Fm.) or younger			<299	Maziviero *et al.* (2013); Crósta *et al.* (2019b)	
100	Tavan Khar Ovoo (Tabun-Khara-Obo)	Mongolia	44°06′N	109°36′E	1.3	Crystalline		Late Triassic to Late Cretaceous		Maximum age based on morphologic expression; likely a few Myr old	150 ± 20?	Masaitis (1999)	
101	Vepriaj	Lithuania	55°06′N	24°36′E	8	Sedimentary		Middle Devonian to Late Jurassic, likely Middle Jurassic			160 ± 5?	Masaitis *et al.* (1980); Masaitis (1999)	
102	Decaturville	United States	37°54′N	92°43′W	6	Mixed		Younger than Pennsylvanian			<323	Offield and Pohn (1977)	
103	Zapadnaya (Bilylivka)	Ukraine	49°44′N	29°00′E	3.2	Crystalline			K-Ar (impact melt rock)		165 ± 6?	Masaitis (1999); Gurov *et al.* (2002)	
104	Obolon’	Ukraine	49°30′N	32°55′E	20	Sedimentary	Iron?	Early Triassic to Middle Jurassic (Bathonian)	K-Ar (impact melt rock)		169 ± 7?	Gurov *et al.* (2009)	
105	Mishina Gora	Russia	58°40′N	28°00′E	2.5	Mixed		Latest Devonian or younger			<360	Masaitis (1999)	
106	Serpent Mound	United States	39°02′N	83°24′W	8	Sedimentary		Tournaisian, Early Mississippian (Cuyahoga Fm.) or younger			<359	Bucher (1936); Reidel and Koucky (1981)	
107	Viewfield	Canada	49°35′N	103°04′W	2.5	Sedimentary		Younger than Mississippian, likely older than Triassic-Jurassic			190 ± 20	Grieve (2006)	
108	São Miguel do Tapuio	Brazil	5°38′S	41°23′W	20	Sedimentary		Late Devonian or younger (Cabeças Fm.)			<382	Crósta *et al.* (2019a, 2019b)	
109	Aorounga	Chad	19°06′N	19°15′E	16	Sedimentary		Late Devonian (?) or younger			≤383	Koeberl *et al.* (2005a)	
110	Gweni-Fada	Chad	17°25′N	21°45′E	22	Sedimentary		Late Devonian (?) or younger			≤383	Koeberl *et al.* (2005a)	
111	Piccaninny	Australia	17°32′S	128°25′E	7	Sedimentary		Frasnian (Late Devonian) or younger			<383	Shoemaker and Shoemaker (1985); Haines (2005)	
112	Puchezh-Katunki	Russia	57°06′N	43°35′E	80	Mixed		Early Triassic to Middle Jurassic	Ar-Ar (impact melt rock)		196–192	Holm-Alwmark *et al.* (2019)	
113	Gow Lake	Canada	56°27′N	104°29′W	5	Crystalline	Iron?		Ar-Ar (impact melt rock)		196.8 ± 9.9	Pickersgill *et al.* (2019b); A. Pickersgill (2019), personal communication	
114	Cloud Creek	United States	43°07′N	106°45′W	7	Sedimentary		Late Triassic (Norian?) to Middle Jurassic (Bathonian?)			∼227 to 166	Stone and Therriault (2003)	
115	Ouarkziz	Algeria	29°00′N	7°33′W	3.5	Sedimentary		Visean, Carboniferous to Paleogene			345–65	Reimold and Koeberl (2014)	
116	Rochechouart	France	45°50′N	0°56′E	23–40	Crystalline	Chondrite? Iron? Stony-iron?		Ar-Ar (impact melt rock)		206.92 ± 0.32	Schmieder *et al.* (2010b); Cohen *et al.* (2017)	
117	Red Wing Creek	United States	47°36′N	103°33′W	9	Sedimentary		Permo-Triassic (Spearfish Fm.) to Bathonian, Middle Jurassic (Piper Fm.)			∼250 to 167	Brenan *et al.* (1975); Koeberl *et al.* (1996)	
118	Wells Creek	United States	36°23′N	87°40′W	12	Sedimentary		Post-Mississippian, older than Late Cretaceous			∼323 to 100	Ford (2015)	
119	Manicouagan	Canada	51°23′N	68°42′W	100	Mixed	No contamination? Chondrite? Achondrite?		U-Pb (ID-TIMS, melt-grown zircon)		215.56 ± 0.05	Hodych and Dunning (1992); Ramezani *et al.* (2005)	
120	Lake Saint Martin	Canada	51°47′N	98°32′W	40	Mixed	No contamination	Devonian to Middle Jurassic	Ar-Ar (impact-melted feldspar and melt rock)		227.8 ± 0.9	Schmieder *et al.* (2014a)	
121	Lumparn	Finland	60°08′20′′N	20°07′30′′E	9	Mixed		Middle Ordovician (Caradocian) or younger			≤458	Merrill (1980); Abels (2003)	
122	Paasselkä	Finland	62°12′N	29°23′E	10	Mixed		Younger than Mesoproterozoic	Ar-Ar (recrystallized feldspar glass and impact melt breccia)		231.0 ± 2.2	Schmieder *et al.* (2010c); Schwarz *et al.* (2015)	
123	Saqqar	Saudi Arabia	29°35′N	38°42′E	34	Sedimentary		Early Devonian (Jauf Fm.) to Late Cretaceous			70–410	Neville *et al.* (2014); Kenkmann *et al.* (2015)	
124	Glover Bluff	United States	43°58′N	89°32'W	8	Sedimentary		Early Ordovician or younger			<485	Read (1983)	
125	Karikkoselkä	Finland	62°13′N	25°14′E	1.5	Crystalline			∼9 Ma (U–Th)/He zircon age	Older paleomagnetic age	∼260 to 230?; ∼9?	Pesonen *et al.* (1999; Schmieder *et al.* (2010a)	
126	Steen River	Canada	59°31′N	117°37′W	25	Mixed		Younger than Late Devonian, older than Mid-Albian	U-Pb (SIMS, zircon in impact melt rock)		∼383 to 108; 132 ± 1.3?	MacLagan (2018); MacLagan *et al.* (2018)	
127	Araguainha	Brazil	16°46′S	52°59′W	40	Mixed		Late Permian to Early Triassic	U-Pb (SHRIMP, LA-ICP-MS, SIMS, monazite and zircon), Ar-Ar (var. lithologies)		254.7 ± 2.5?; 259 ± 5?; 251.5 ± 2.9?	Tohver *et al.* (2012); Erickson *et al.* (2017); Hauser *et al.* (2019)	
128	Glikson	Australia	23°59′S	121°34′E	19	Sedimentary		Paleozoic			<508 ± 5	Macdonald *et al.* (2005)	
129	Kursk	Russia	51°40′N	36°00′E	6	Sedimentary		Early Carboniferous to Middle Jurassic			359–163	Masaitis (1999)	
130	Gosses Bluff (Tnorala)	Australia	23°50′S	132°19′E	22	Sedimentary		Late Devonian or younger	Ar-Ar minimum age (impact melt rock)		∼383 to 165	Schmieder, Tohver and Jourdan (unpublished data); Haines (2005)	
131	Douglas (Sheep Mountain) (Field)	Wyoming, United States	42°40′N	105°28′W	0.15	Sedimentary		Early Permian (Uppermost Casper Fm.)			∼280	Kastning and Huntoon (1996); Kenkmann *et al.* (2018)	
132	Ternovka (Terny)	Ukraine	48°01′N	E33°05′	16–19	Mixed	Chondrite?		K-Ar (feldspar and mica)		280 ± 10	Val'ter *et al.* (1981)	
133	West Clearwater Lake	Canada	56°13′N	74°30′W	36	Mixed	No contamination		Ar-Ar (impact melt rock)		286.2 ± 2.6	Bottomley *et al.* (1990); Schmieder *et al.* (2015a)	
134	Luizi	Democratic Republic of Congo	10°10′S	27°55′E	17	Sedimentary		Late Neoproterozoic or younger			≤573	Master *et al.* (2001); Ferrière *et al.* (2011)	
135	Elbow	Canada	50°59′N	106°43′W	8	Sedimentary		Middle Devonian to pre-Jurassic			393–201	Grieve (2006)	
136	Saarijärvi	Finland	65°17′N	28°25′E	1.5	Crystalline		Ediacaran to Early Cambrian or younger			<600–520	Öhman (2007)	
137	Dobele	Latvia	56°35′N	23°15′E	4.5	Sedimentary		Early Carboniferous to Late Permian			359–252	Masaitis (1999)	
138	West Hawk Lake	Canada	49°46′N	95°11′W	2.44	Crystalline		Younger than ∼2.22 Ga		Fission track	351 ± 20?	Grieve (2006)	
139	Siljan	Sweden	61°02′N	14°52′E	52	Mixed			Ar-Ar (impact melt rock)		380.9 ± 4.6	Jourdan and Reimold (2012)	
140	Flynn Creek	United States	36°17′N	85°40′W	3.8	Sedimentary		Late Devonian (conodonts)			∼382 Ma	Schieber and Over (2005)	
141	Kaluga	Russia	54°30′N	36°15′E	15	Mixed		Middle Devonian			∼394 to 383	Masaitis (1999, 2002)	
142	Nicholson Lake	Canada	62°40′N	102°41′W	12.5	Mixed	Achondrite		U-Pb (impact-heated apatite)		387 ± 5	McGregor *et al.* (2018)	
143	Crooked Creek	United States	37°50′N	91°23′W	7	Sedimentary		Early Ordovician to pre-Pennsylvanian			485–323	Snyder and Gerdemann (1965)	
144	Lac Couture	Canada	60°08′N	75°20′W	8	Crystalline			Ar-Ar (impact melt rock)		429 ± 25	Bottomley *et al.* (1990), recalculated	425 ± 25
145	Tunnunik (Prince Albert)	Canada	72°27′N	113°54′W	25	Sedimentary				Paleomagnetic age	∼450 to 430	Lepaulard *et al.* (2019)	
146	Charlevoix (Malbaie)	Canada	47°32′N	70°18′W	54	Mixed		Katian or younger (Neuville Fm.)	U-Pb (zircon in impact melt rock)		∼453 to 430	Whitehead *et al.* (2003); Schmieder *et al.* (2019)	
147	Ilyinets	Ukraine	49°08′N	29°11′E	8.5	Mixed	Iron?		Ar-Ar (impact melt breccia)		445 ± 10	Pesonen *et al.* (2004)	
148	Glasford	United States	40°36′N	89°47′W	4	Sedimentary		“Early Cincinnatian” ( = Katian)			∼453 to 445	Buschbach and Ryan (1963)	
149	Pilot	Canada	60°17′N	111°01′W	6	Crystalline			Ar-Ar (impact melt rock)		450 ± 2	Bottomley *et al.* (1990), recalculated	445 ± 2
150	Slate Islands	Canada	48°40′N	87°00′W	30	Mixed			Ar-Ar (impact melt rock)		∼450	Sharpton *et al.* (1997); Grieve (2006)	
151	Calvin	United States	41°50′N	85°57′W	8.5	Sedimentary		Late Ordovician			458–444	Milstein (1994)	
152	La Moinerie	Canada	57°26′N	66°37′W	8	Crystalline		Ordovician, pre-Silurian	U-Pb (LA-ICP-MS on apatite)		453 ± 5	McGregor *et al.* (2019)	
153	Brent	Canada	46°05′N	78°29′W	3.8	Crystalline	Chondrite (L or LL?)	Early Caradoc ( = Sandbian), conodont and chitinozoan biostratigraphy			458–453	Lozej and Beales (1975); Grahn and Ormö (1995)	
154	Kärdla	Estonia	58°59′N	22°40′E	4	Mixed		Transition *A. curvata/Lagenochitina dalbyensis* zone ( = late Sandbian), likely slightly older than Lockne			455 ± 1	Grahn *et al.* (1996)	
155	Lockne	Sweden	63°00′N	14°48′E	7.5–14	Mixed		Lower *L. dalbyensis* zone ( = late Sandbian), likely slightly younger than Kärdla			455 ± 1	Grahn *et al.* (1996); Grahn (1997); Ormö *et al.* (2014)	
156	Målingen	Sweden	62°55′N	14°33.84′E	0.7	Mixed		Lower *L. dalbyensis* zone ( = late Sandbian), contemporary with Lockne			455 ± 1	Ormö *et al.* (2014); cf. Grahn *et al.* (1996)	
157	Tvären	Sweden	58°46′N	17°25′E	2	Mixed		*Lagenochitina stentor* zone ( = early Sandbian)			∼458	Ormö (1994); Grahn *et al.* (1996)	
158	East Clearwater Lake	Canada	56°05′N	74°07′W	26	Crystalline	Chondrite (L or LL?)		Ar-Ar (impact melt rock)		470–460	Bottomley *et al.* (1990); Schmieder *et al.* (2015a)	
159	Hummeln	Sweden	57°22′04′′N	16°14′56′′E	1.2	Mixed	Chondrite	Upper *C. regnelli* zone ( = early Darriwilian) (chitinozoans)			∼465	Alwmark *et al.* (2015), after Grahn *et al.* (1996)	
160	Granby	Sweden	58°25′N	14°56′E	3	Sedimentary		Lower *C. regnelli* zone ( = early Darriwilian) (chitinozoans)			∼466	Alwmark (2009), after Grahn *et al.* (1996)	
161	Decorah	United States	43°19′N	91°46′WW	5.5	Sedimentary		Early–Middle Darriwilian (Middle Ordovician), pre-Winneshiek Shale			∼467 to 464	Bergström *et al.* (2018); French *et al.* (2018)	
162	Ames	United States	36°15′N	98°10′W	16	Mixed		Early to Middle Ordovician (Floian to Darriwilian)			∼478 to 458	Koeberl *et al.* (2001)	
163	Rock Elm	United States	44°43′N	92°14′W	6	Sedimentary		Early to Middle Ordovician			∼485 to 458	Cordua (1985); French *et al.* (2004)	
164	Lawn Hill	Australia	18°40′S	138°39′E	18	Mixed			Ar-Ar (impact melt breccia)		476 ± 8	Darlington *et al.* (2016), recalculated	472 ± 8
165	Ramgarh	India	25°20′N	76°37′E	10	Sedimentary		Neoproterozoic to Middle Jurassic (Callovian)			∼750 to 165; ∼165?	Ray *et al.* (2003); Kenkmann *et al.* (2019)	
166	Carswell	Canada	58°27′N	109°30′W	39	Mixed			Ar-Ar (adularia in impact melt rock)		481.5 ± 0.8 Ma	Alwmark *et al.* (2017)	
167	Newporte	United States	48°58′N	101°58′W	3	Sedimentary		Late Cambrian to Early Ordovician (Deadwood. Fm.)			∼500 to 480	Koeberl and Reimold (1995)	
168	Mizarai	Lithuania	54°00′N	23°54′E	5	Mixed		Late Cambrian to Early Ordovician			∼520 to 480	Masaitis (1999); Abels *et al.* (2002)	
169	Ritland	Norway	59°14′N	6°26′E	2.7	Mixed		Early to Middle Cambrian			∼540 to 500	Riis *et al.* (2011)	
170	Neugrund	Estonia	59°20′N	23°31′E	20	Crystalline		Early Cambrian			∼540 to 530	Suuroja and Suuroja (2000)	
171	Gardnos	Norway	60°39′N	9°00′E	5	Crystalline	Chondrite? Nonmagmatic iron?	Neoproterozoic to Cambrian	U-Pb (zircon in melt breccia)		546 ± 5?	Kalleson *et al.* (2009)	
172	Holleford	Canada	44°28′N	76°38′W	2.35	Crystalline		Latest Proterozoic to Early Paleozoic?			550 ± 100	Grieve (2006)	
173	Acraman	Australia	32°01′S	135°27′E	40–90	Crystalline (dacite)	Chondrite	Distal ejecta in Ediacaran Bunyeroo Fm.			∼635 to 541	Williams and Gostin (2005); Schmieder *et al.* (2015b) and references therein	
174	Sääksjärvi	Finland	61°24′N	22°24′E	6	Crystalline	Stony-iron? Iron? Chondrite?		U–Pb (zircon from impact melt rock)		602 ± 17	Mänttäri *et al.* (2004)	
175	Strangways	Australia	15°12′S	133°35′E	25	Mixed	Achondrite		Ar-Ar (impact melt rock)		657 ± 43	Spray *et al.* (1999), recalculated	646 ± 42
176	Beaverhead	United States	44°36′N	113°00′W	60	Mixed			Ar-Ar (impact melt rock)		900–470	Hargraves *et al.* (1994)	
177	Jänisjärvi	Russia	61°58′N	30°55′E	14	Crystalline			Ar-Ar (impact melt rock)		687 ± 5	Jourdan *et al.* (2012) after Jourdan *et al.* (2008)	
178	Goyder	Australia	13°29′S	135°02′E	3	Sedimentary		Mesoproterozoic or younger			∼1325 to 150	Haines (2005)	
179	Spider	Australia	16°44′S	126°05′E	13	Sedimentary		Paleoproterozoic (Pentecost Sandstone) or younger; younger than Yampi Orogeny; older than Ediacaran (Marinoan glaciation)			∼900 to 580	Abels (2005)	
180	Île Rouleau	Canada	50°41′N	73°53′W	4	Sedimentary		Paleoproterozoic or younger			<1800	Grieve (2006)	
181	Santa Fe	United States	35°45′N	105°56′W	6–13	Crystalline			U–Pb (zircon)		1472–350	Montalvo *et al.* (2018)	
182	Matt Wilson	Australia	15°30′S	131°11′E	7.5	Sedimentary		Mesoproterozoic (Jasper Gorge Sandstone) or younger			<1344	Haines (2005); Kenkmann and Poelchau (2009)	
183	Shoemaker (Lake Teague)	Australia	25°52′S	120°53′E	30	Mixed		Proterozoic (Teague Granite) or younger	Minimum K-Ar alteration age (illite)		∼1300 to 568	Pirajno *et al.* (2003)	
184	Summanen	Finland	62°39.0′N	25°22.5′CE	2.6	Crystalline		Paleoproterozoic or younger			<1880	Plado *et al.* (2018)	
185	Cleanskin	Australia	18°10′S	137°56′E	15	Sedimentary		Mesoproterozoic to Early Cambrian			∼1400 to 520	Haines *et al.* (2012)	
186	Foelsche	Australia	16°40′S	136°47′E	6	Sedimentary		Mesoproterozoic to Early Cambrian			∼1496 to 520	Haines and Rawlings (2002)	
187	Iso-Naakkima	Finland	62°11′57′′N	27°07′59′′E	3	Sedimentary		Meso- to Neoproterozoic			∼1200 to 900	Elo *et al.* (1993); Pesonen *et al.* (1996a)	
188	Kamenetsk	Ukraine	47°46′N	32°21′E	1.2	Crystalline		Paleoproterozoic to Late Miocene			2100–5	Gurov *et al.* (2017)	
189	Woodleigh	Australia	26°05′S	114°43′E	60	Mixed		Proterozoic (Dalgaringa Supersuite)—Bajocian, Middle Jurassic			2005–168	Renne *et al.* (2002); Sheppard *et al.* (2010)	
190	Kelly West	Australia	19°56′S	133°57′E	10	Crystalline		Proterozoic, likely Neoproterozoic			∼1640 to 550	Haines (2005)	
191	Amelia Creek	Australia	20°51′S	134°53′E	20	Mixed		Paleoproterozoic to Late Neoproterozoic			1640–600	Haines (2005)	
192	Keurusselkä	Finland	62°08′N	24°36′E	30	Crystalline			Ar-Ar (impact melt breccia)		1151 ± 10	Schmieder *et al.* (2016a)	
193	Liverpool	Australia	12°24′S	134°03′E	1.6	Sedimentary		Paleo- to Neoproterozoic?			∼1870 to 541?	Shoemaker and Shoemaker (1997); Shoemaker *et al.* (2005)	
194	Söderfjärden	Finland	63°00′N	21°35′E	6.6	Crystalline			Ar-Ar (melt breccia)		∼1880 to 640	Schmieder *et al.* (2014b)	
195	Suvasvesi South	Finland	62°24′N	28°12′E	3.8	Crystalline			Ar-Ar (impact melt rock)		∼710 to 1880	Schmieder *et al.* (2016b); Schwarz *et al.* (2016a)	
196	Presqu'île	Canada	49°43′N	78°48′W	24	Crystalline		Neoarchean or younger			<2729	Higgins and Tait (1990)	
197	Sudbury	Canada	46°36′N	81°11′W	200	Crystalline			U-Pb (CA-TIMS, melt-grown zircon in felsic norite)		1849.53 ± 0.21	Krogh *et al.* (1984); Davis (2008)	
198	Vredefort	South Africa	27°00′S	27°30′E	250	Crystalline	Chondrite?		U-Pb (CA-TIMS, melt-crystallized zircon)		2023 ± 4	Kamo *et al.* (1996)	
199	Dhala	India	25°18′N	78°08′E	11	Mixed	Chondrite	Younger than Bundelkhand Craton, older than Vindhyan Supergroup			∼2500 to 1700	Pati *et al.* (2008, 2010)	
200	Yarrabubba	Australia	27°10′S	119°50′E	30–70	Crystalline			U-Pb (monazite and zircon in impact melt rock)		2229 ± 5	Fletcher and McNaughton (2002); Erickson *et al.*, (2019a, 2019b)	

Sorting by “numerical” age (not listed for stratigraphic maximum ages). A stratigraphic age of ≤573 Ma (Luizi) can alternatively be written as a numerical value of 287 ± 287 Ma and is then listed before a seemingly younger age, such as 455 ± 1 Ma (Lockne). In such cases, the more conservative stratigraphic maximum/minimum age notation is preferred over the numerical value.

^a^ Type of target rock largely taken from the *Earth Impact Database* (as of 2018; now offline) and Osinski and Grieve (2019).

^b^ Type of impactor taken from the *Earth Impact Database* (2018) and the literature, including Palme *et al.* (1978, 1979, 1981), Morgan *et al.* (1979), Evans *et al.* (1993), Schmidt and Pernicka (1994), Schmidt *et al.* (1997), Koeberl (1998), Maier *et al.* (2006), Tagle and Hecht (2006), Koeberl *et al.* (2007a), Tagle *et al.* (2009), Goderis *et al.* (2009, 2013), Koeberl (2014), Magna *et al.* (2017), Pati *et al.* (2017), Buchner *et al.* (2018), and Mougel *et al.* (2019), and references in those articles.

^c^ Recalculated ages calculated using the ArAR tool of Mercer and Hodges (2016).

^d^ (Temporary) impact penetration hole.

^e^ Impact pit(s).

^f^ Field of impact craters (higher energy) together with impact pits and/or funnels (lower energy).

CA-TIMS = chemical abrasion thermal ionization mass spectrometry; ID-TIMS = isotope dilution thermal ionization mass spectrometry; LA-ICP-MS = laser ablation inductively coupled plasma mass spectrometry; SHRIMP = sensitive high-resolution ion microprobe; SIMS = secondary ion mass spectrometry.

**Table 2.** List of Terrestrial Impact Deposits (Impact Ejecta; Breccias), Select Age Constraints, and Recommended Impact Ages, Sorted by Age

	Impact ejecta and deposits	Country	Latitude	Longitude	Diameter (km)	Type of impactor^a^	Stratigraphic age constraints	Radioisotopic age constraints	Other age constraints	Recommended age (Ma)	Recommended age reference^b^	Pre-recalculation age (Ma)
1	Rio Cuarto (fresh)	Argentina				Chondrite (H?)	Holocene	Ar-Ar (glass)	Source crater uncertain	∼0.004 to 0.010; 0.006 ± 0.002	Schultz *et al.* (2004, 2006)	
2	Rio Cuarto (old)	Argentina				Chondrite (H?)	Pleistocene	Ar-Ar (glass)	Source crater uncertain	0.115 ± 0.026; 0.57 ± 0.2	Schultz *et al.* (2004, 2006), recalculated; Bland *et al.* (2002)	0.114 ± 0.026
3	Centinela del Mar	Argentina					Pleistocene	Ar-Ar (glass)	Source crater unknown	0.232 ± 0.030; 0.449 ± 0.021	Schultz *et al.* (2004, 2006), recalculated	0.230 ± 0.030; 0.445 ± 0.021
4	Belize impact glass	Belize					Pleistocene	Ar-Ar (glass)	Source crater unknown (Pantasma?)	0.769 ± 0.016	Schwarz *et al.* (2016b)	
5	Australasian Tektites	Semiglobal				Chondrite? Achondrite?	Pleistocene	Ar-Ar (glass)	Source crater unknown	0.7881 ± 0.0028	Jourdan *et al.* (2019)	
6	Darwin Glass	Australia				Inconclusive	Pleistocene	Ar-Ar (glass)	Associated with “Darwin crater”?	0.828 ± 0.007	Lo *et al.* (2002), recalculated	0.816 ± 0.007
7	Ivory Coast tektites	Cote d'Ivoire, Atlantic				Ordinary chondrite?	Pleistocene	Ar-Ar (Ivory Coast tektites)	From Bosumtwi impact	1.13 ± 0.10	Jourdan (2012), after Koeberl *et al.* (1997b)	
8	Eltanin spherules	Southern Ocean				Mesosiderite	Pleistocene		From Eltanin impact into water column	∼2.51 ± 0.07	Gersonde *et al.* (1997); Frederichs *et al.* (2002)	
9	Mar de Plata	Argentina					Pliocene	Ar-Ar (glass)	Source crater unknown	3.37 ± 0.10	Schultz *et al.* (1998, 2004, 2006), recalculated	3.33 ± 0.10
10	Bahía Blanca	Argentina					Late Miocene	Ar-Ar (glass)	Source crater unknown	5.38 ± 0.05	Schultz *et al.* (2004, 2006), recalculated	5.33 ± 0.05
11	Chasico	Argentina					Late Miocene	Ar-Ar (glass)	Source crater unknown	9.32 ± 0.09	Schultz *et al.* (2004, 2006), recalculated	9.23 ± 0.09
12	Atacamaites	Chile				Iron?	Likely Neogene		Source crater unknown	Unknown	Koeberl *et al.* (2019)	
13	Central European Tektites	Czech Republic, Austria, Germany, Poland				No contamination (achondrite?)	Middle Miocene	Ar-Ar (moldavite tektites)	From Ries impact	14.808 ± 0.038	Schmieder *et al.* (2018a, 2018b)	
14	Uruguaites	Uruguay					Eocene or younger?		Source crater unknown	<56	Ferrière *et al.* (2017)	
15	Libyan Desert Glass	Egypt				Chondrite			Glass fission track	28.5 ± 0.8	Bigazzi and De Michele (1996)	
16	North American tektites	United States				Chondrite	Late Eocene	Ar-Ar (impact melt rock)	From Chesapeake impact	34.86 ± 0.32	Assis Fernandes *et al.* (2019)	
17	Clinopyroxene spherules	Semiglobal				Chondrite	Late Eocene	Ar-Ar (impact melt rock)	From Popigai impact	35.7 ± 0.2	Bottomley *et al.* (1997); Whitehead *et al.* (2000)	
18	Solin	Croatia					Late Eocene (Priabonian; E14/15 zone)		Source crater unknown	∼38 to 34	Marjanac *et al.* (2006)	
19	Paleogene/Eocene boundary microtektites	Atlantic					Paleocene/Eocene boundary		Source crater unknown	∼56	Schaller *et al.* (2016); Schaller and Fung (2018)	
20	Nuussuaq (Disko) spherules	Greenland					Paleocene		Source crater unknown	∼60	Robin *et al.* (1996); Glass and Simonson (2012)	
21	K/T (K/Pg) boundary ejecta	Worldwide				Carbonaceous chondrite	Cretaceous/Paleogene boundary	Ar-Ar (impact glass spherules)	From Chicxulub impact	66.052 ± 0.043	Renne *et al.* (2013, 2018); Sprain *et al.* (2015, 2018); Clyde *et al.* (2016)	
22	Tookoonooka distal ejecta horizon	Australia					Barrêmian/Aptian boundary		From Tookoonooka impact	125 ± 1	Bron and Gostin (2012)	
23	Raeside breccia	Australia	28°47′S	120°58′E	Unknown		Permian—Neogene		Proximal ejecta?	290–34	Glikson *et al.* (2016a)	
24	Late Triassic glauconite spherules	United Kingdom					Late Triassic (Norian)		From Manicouagan impact	215.56 ± 0.05	Walkden *et al.* (2002); Kirkham (2003)	
25	Qidong spherules	China					Late Devonian (Early Famennian); *P. crepida* conodont zone		Source crater unknown	∼370	Wang and Chatterton (1993)	
26	Senzeille/Hony microtektites	Belgium					Late Devonian (right above Frasnian/Famennian boundary)		Source crater unknown	∼372	Claeys *et al.* (1992); Glass and Simonson (2012, 2013)	
27	Alamo breccia	United States	37°30′N	116°30′W	Unknown		Middle Frasnian, Late Devonian		Source crater tectonically recycled/buried?	382–372	Pinto *et al.* (2008); Morrow *et al.* (2009)	
28	Hallen ejecta layer	Sweden					Late Sandbian (Late Ordovician)		From Lockne–Målingen impact	∼455	Sturkell *et al.* (2000)	
29	Osmussaar Breccia	Estonia	59°18′N	23°21′E	Unknown		Middle Ordovician (Early Darriwilian)		Source crater unknown	∼466	Alwmark *et al.* (2010)	
30	Vakkejokk Breccia	Sweden	68°22′N	19°14′E	Unknown		Middle Cambrian		Source crater hidden underneath mountain	∼521 to 514	Ormö *et al.* (2017)	
31	Acraman-Bunyeroo horizon	Australia				Chondrite	Ediacaran		From Acraman impact	∼635 to 541	Schmieder *et al.* (2015b) and references therein	
32	Stac Fada Member	Scotland, United Kingdom			Unknown	Chondrite	Mesoproterozoic	Ar-Ar (authigenic K-feldspar)	Source crater hidden	1177 ± 5	Parnell *et al.* (2011)	
33	Lake Superior/Michigan ejecta	United States					Paleoproterozoic	U-Pb (CA-TIMS, melt-grown zircon in felsic norite)	From Sudbury impact	1849.53 ± 0.21	Addison *et al.* (2005); Cannon *et al.* (2010)	
34	Grænsesø	Greenland				Carbonaceous chondrite	Paleoproterozoic		Source crater unknown	∼1990	Glass and Simonson (2012, 2013); Huber *et al.* (2019)	
35	Zaonega spherules	Russia				Carbonaceous chondrite	Paleoproterozoic		From Vredefort impact?	∼2050 to 1975	Huber *et al.* (2014)	
36	Dales Gorge; Kuruman	Western Australia; South Africa				Ordinary chondrite? Enstatite chondrite?	Paleoproterozoic		Source crater unknown	2495 ± 16	Simonson *et al.* (2009); Glass and Simonson (2012, 2013)	
37	Bee Gorge; Paraburdoo; Reivilo; Wittenoom	Western Australia; South Africa				Chondrite?	Neoarchean		Source crater unknown	2541 ± 18	Simonson *et al.* (2009); Hassler *et al.* (2011); Glass and Simonson (2012, 2013)	
38	Jeerinah; Carawine; Monteville	Western Australia; South Africa				Ordinary chondrite? Enstatite chondrite?	Neoarchean		Source crater unknown	2629 ± 5	Rasmussen *et al.* (2005); Simonson *et al.* (2009); Glass and Simonson (2012, 2013)	
39	S5 (Barberton)	South Africa					Paleoarchean		Source crater unknown	3234 ± 5	Lowe and Byerly (2010); Glass and Simonson (2012, 2013); Lowe *et al.* (2014)	
40	S4 (Barberton)	South Africa				Carbonaceous chondrite	Paleoarchean		Source crater unknown	∼3243	Lowe *et al.* (2003, 2014); Glass and Simonson (2012, 2013)	
41	S3 (Barberton)	South Africa				Carbonaceous chondrite	Paleoarchean		Source crater unknown	∼3243	Lowe *et al.* (2003, 2014); Glass and Simonson (2012, 2013)	
42	S2 (Barberton)	South Africa				Carbonaceous chondrite	Paleoarchean		Source crater unknown	∼3260	Lowe *et al.* (2003, 2014); Glass and Simonson (2012, 2013)	
43	S6 (Barberton)	South Africa					Paleoarchean		Source crater unknown	∼3330	Lowe and Byerly (2010); Glass and Simonson (2012, 2013); Lowe *et al.* (2014)	
44	S7 (Barberton)	South Africa					Paleoarchean		Source crater unknown	3416 ± 5	Kröner *et al.* (1991); Lowe and Byerly (2010); Glass and Simonson (2012, 2013); Lowe *et al.* (2014)	
45	Marble Bar	Western Australia					Paleoarchean		Source crater unknown	∼3460	Glikson *et al.* (2016b)	
46	S1 (Barberton); Warrawoona	South Africa; Western Australia					Paleoarchean		Source crater unknown	3470 ± 2	Byerly *et al.* (2002); Lowe *et al.* (2003, 2014); Glass and Simonson (2012, 2013)	

^a^ Type of impactor taken from Meisel *et al.* (1990), Koeberl (1997), McDonald (2002), Kyte *et al.* (2003, 2011), Tagle and Hecht (2006), Simonson *et al.* (2009), Goderis *et al.* (2012, 2013, 2017), Glass and Simonson (2013), Koeberl (2014), Mougel *et al.* (2017), Folco *et al.* (2018), and references in those articles.

^b^ Recalculated ages calculated using the ArAR tool of Mercer and Hodges (2016).

The smallest geologic features on Earth's surface produced by impact, usually only a few meters wide and commonly associated with surviving meteorite fragments, are (fields of) penetration funnels, pits, and small craters that form at relatively low, atmosphere-decelerated (ballistic) impact velocities (*e.g*., Melosh, 1989; Beauford, 2015). Some of the impact structures listed in this article belong to that type of low-energy impact feature (*e.g*., the crater-like pits produced during the fall of the Imilac pallasite in Chile, or the temporary Chalyabinsk ice-penetration hole, which we chose to include in the present listing). Hypervelocity impacts of larger meteoroids, at much higher incoming velocities, produce craters that show different morphologies with increasing size (*e.g*., Melosh, 1989; French, 1998). A textbook example of a well-preserved simple, bowl-shaped impact crater associated with its ejecta blanket is the ∼1.2 km-diameter Meteor Crater (*a.k.a.* Barringer Meteorite Crater) in Arizona (Shoemaker, 1960; Kring, 2017b) (Fig. 1). Earth's impact craters larger than ∼2 to 4 km in diameter are of complex morphology and structure, such as the ∼3.8 km-diameter Steinheim Basin in Germany characterized by a pronounced central peak (uplift) and the ∼25 km-diameter Nördlinger Ries with an ∼10 km-wide inner ring of uplifted target rock and a well-preserved blanket of proximal impact ejecta surrounding the crater (*e.g*., Stöffler *et al.*, 2002, 2013; Kring, 2005; Schmieder and Buchner, 2013). The 180 km-diameter Chicxulub crater on the Yucatán Peninsula in Mexico is a peak-ring basin similar in morphology and structure to the Schrödinger Basin on the Moon (Kring, 1995; Kring *et al.*, 2016, 2017a; Morgan *et al.*, 2016). The deeply eroded Vredefort impact structure in South Africa, probably ∼250 to 300 km in original diameter, may represent the remnants of a terrestrial multiring basin (*e.g*., Melosh, 1989; Spudis, 1993; Therriault *et al.*, 1997; French, 1998).

To assess the temporal distribution of impact events and calculate impact rates as an expression of the impact flux through time, different geochronologic techniques have been developed and applied. These include, first, crater counting and the calculation of isochrons based on the crater size–frequency distribution for the Moon, Mars, and other planetary bodies characterized by a crater production record (*e.g*., Hartmann and Neukum, 2001; this technique is not applicable to the geologically active Earth); second, stratigraphic age constraints (*e.g*., Koeberl *et al.*, 2001; Lindström *et al.*, 2005; Schmieder and Buchner, 2008); third, isotopic age determinations using the U–Pb, Ar–Ar (K–Ar), Rb–Sr, and (U–Th)/He geo-/thermochronometers and/or the ^14^C method with impact lithologies sampled in natural outcrop or drillings on Earth, in meteorites, or samples returned from space missions (*e.g*., Tera *et al.*, 1974; Bottomley *et al.*, 1990; Deutsch and Schärer, 1994; Jourdan *et al.*, 2009, 2012); and, finally, methods other than those mentioned above. We here predominantly focus on the stratigraphic and isotopic methods. Due to improvements in U–Pb (*e.g*., chemical abrasion thermal ionization mass spectrometry [CA-TIMS]) (Schoene, 2014; Kenny *et al.*, 2019a), secondary ion mass spectrometry (SIMS) (Kenny *et al.*, 2019b), and ^40^Ar–^39^Ar geochronologic instrumentation and methods (*e.g*., Renne *et al.*, 2010, 2011, 2013; Sprain *et al.*, 2015; Schmieder *et al.*, 2018a), the most precisely constrained “impact ages” today come with uncertainties on the thousands-of-years (ka, kyr) level.

This article provides a current (as of September 2019) summary of predominantly stratigraphic and isotopic recommended ages for proven impact structures and deposits on Earth. Structures and deposits of likely but, to some degree, uncertain impact origin (*e.g*., numerous oblong depressions near Rio Cuarto in Argentina; Schultz and Lianza, 1992; cf. Cione *et al.*, 2002; Reimold *et al.*, 2018; Crósta *et al.*, 2019c; the recently reported Hiawatha “impact crater” in Greenland; Kjær *et al.*, 2018; and enigmatic glass deposits such as the Edeowie glass found in South Australia; Haines *et al.*, 2001; glasses found near Dakhleh, Egypt; Osinski *et al.*, 2008; and the Pica glass found in the Atacama Desert of Chile; Roperch *et al.*, 2017) are, therefore, not included. Likewise, the 1908 Tunguska airburst event in Russia, which seemingly did not produce any geologic feature other than uprooted trees, is not listed here (*e.g*., Kulik, 1940; Krinov, 1960). The present article does not intend to be the latest reference pertaining to the formation of simple and complex impact craters, their impact ejecta, and the physical aspects of the cratering process (*e.g*., Melosh, 1989; Melosh and Ivanov, 1999; Osinski *et al.*, 2011, 2012; Kenkmann *et al.*, 2012), the petrology of impactites (rocks produced or modified by impact) (*e.g*., French, 1998; Stöffler and Grieve, 2007; Grieve and Therriault, 2012), or the verification of impact structures through the identification of macro- and microscopic shock-metamorphic features (*e.g*., shatter cones and shocked quartz and zircon grains) (French, 1998; French and Koeberl, 2010; Ferrière and Osinski, 2012). For details about the more specific geologic features of terrestrial impact structures, we refer the reader to a number of review articles that summarize the impact cratering record of each continent on Earth, such as the works of Grieve (2006) for Canada in North America, Reimold *et al.* (2018) and Crósta *et al.* (2019b) for South America, Schmieder and Buchner (2013) for Europe, Reimold and Koeberl (2014) and Chabou (2019) for Africa and the Arab World, respectively, Masaitis (1999) and Reimold *et al.* (2008) for Russia and Asia, and Haines (2005) for Australia. [Somewhat surprisingly, there is currently no up-to-date review of the impact cratering record of the United States, and Walter H. Bucher's (1936) early work on the country's “cryptoexplosion structures” probably remains the most recent systematic review of its kind; however, many impact structures in the United States were included in the more general listings of Freeberg (1969), Classen (1977), and Grolier (1985), and a website project maintained by Beauford (2019) provides basic information and the relevant literature for almost all impact structures and crater fields recognized in the country.] Nor does this relatively short summary provide an in-depth explanation and discussion of the isotopic methods commonly used to determine impact ages, such as the U–Pb and Ar–Ar geochronometers. In this context, we recommend the comprehensive summaries on the U–Pb technique by, for example, Corfu (2013) and Schoene (2014), and on the Ar–Ar (and K–Ar) method by McDougall and Harrison (1999) and Kelley (2002). Previous U–Pb, Ar–Ar, and Rb–Sr geochronologic work on several terrestrial impact structures includes that of Bottomley *et al.* (1990) and Deutsch and Schärer (1994), from which much was learned regarding how different geochronometers behave with different types of impact crater materials analyzed. This summary builds upon that previous work, including critical evaluations of Earth's impact crater ages that ensued (Jourdan *et al.*, 2009, 2012; Jourdan, 2012). It should serve as a robust geochronologic database and a backbone for ongoing and future studies that make use of Earth's impact crater ages for, for example, statistical calculations and cratering flux models (*e.g*., Mazrouei *et al.*, 2019). Such studies have, in part, relied on a flawed representation of the terrestrial impact cratering record with partly inaccurate ages as input parameters (*e.g*., Telecka and Matyjasek, 2011; and the recently published *Encyclopedic Atlas of Terrestrial Impact Craters* of Flamini *et al.*, 2019 that lists numerous inaccurate impact ages), inevitably compromising the validity and significance of their conclusions (see also discussions in Miljković *et al.*, 2013, 2014; Schmieder *et al.*, 2014c; Rampino and Caldeira, 2015; Meier and Holm-Alwmark, 2017). Finally, this work presents a referenced source for current best-estimate ages that can be listed in online impact databases, such as the *Earth Impact Database* (hosted at the University of New Brunswick, Fredericton, Canada), which has recently been complemented by the database *Impact Earth* maintained by Osinski and Grieve (2019).

## 2. Data and Methods

Stratigraphic, isotopic, and additional age constraints are predominantly sourced from the primary literature, highlighting the work that led to the establishment of the (currently) preferred age for any particular impact event. Some ages are taken from summary articles (*e.g*., Grieve, 2006). Impact ages are grouped into three main categories: (1) stratigraphic age constraints; (2) isotopic ages, including U–Pb, Ar–Ar, K–Ar, Rb–Sr, (U–Th)/He, and ^14^C ages (while considering ages obtained using the high-temperature U–Pb and Ar–Ar geochronometers are usually preferred); and (3) age constraints other than the ones mentioned above.

### 2.1. Stratigraphic ages

The determination of relative stratigraphic ages, by superposition, can be applied to all impact structures on Earth and elsewhere, where the age of the host rock is to some degree constrained. Every impact structure has a target rock that the impacting body penetrated and, through simple geologic cross-cutting relationships, the youngest rock units affected by the impact provide a maximum (oldest possible) age for the impact. In turn, the oldest undisturbed rocks that fill the crater after its formation, commonly crater lake sediments in continental paleosettings, constrain the minimum (youngest possible) impact age. Some terrestrial impact crater ages are only very imprecisely constrained by the age of the impacted target rock as a maximum age (*e.g*., the <1800 million years [Ma, Myr] Île Rouleau impact structure, Québec, Canada) (Grieve, 2006). Sometimes, the stratigraphic age for an impact can only be bracketed within several hundred million years, as in the case of the 12 km-diameter Wells Creek impact structure in Tennessee (*e.g*., Wilson, 1953; Ford *et al.*, 2012; Ford, 2015, and references therein). The crater must be younger than Mississippian (∼323 Ma) and older than Late Cretaceous (∼100 Ma) (see Cohen *et al.*, 2013, for current stratigraphic age values), suggesting a “best-estimate” age of ∼211 ± 111 Ma and a relative error on the age of >100% (the commonly published age is 200 ± 100 Ma) (*e.g*., Grieve, 2001a). However, other stratigraphically constrained impact ages are remarkably precise, such as that of the ∼14 km-wide marine Lockne crater in Ordovician rocks of Central Sweden. There, the impact age is precisely constrained to be 455 Ma plus and minus a few hundred thousand years, because both the youngest preimpact and oldest postimpact sediments lie in the late Sandbian (early Caradocian) lower *Lagenochitina dalbyensis* chitinozoan microfossil zone studied in great detail (Grahn *et al.*, 1996; Grahn, 1997; Ormö *et al.*, 2014). The stratigraphic method equally applies to impact ejecta layers.

### 2.2. Isotopic ages

**FIG. 3.**
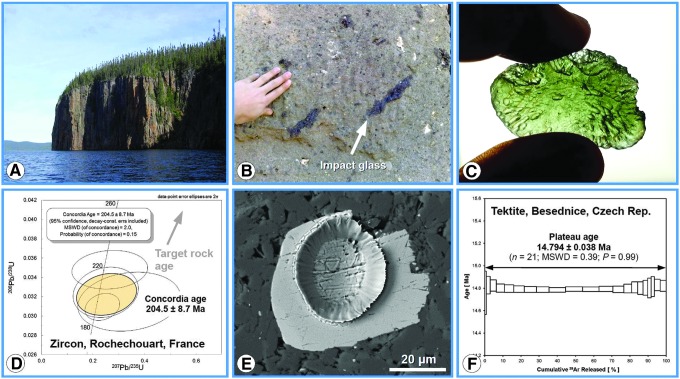
Impact crater materials commonly used for geochronologic analysis and two exemplary results. **(A)** Approximately 100 m-tall cliff of the impact melt sheet at the Manicouagan impact structure, Québec, Canada (Baie Memory Entrance Island, photo taken by M. Schmieder in summer 2006). This type of impact melt rock is suitable for whole-rock Ar–Ar analysis and usually contains minerals (*e.g*., zircon) that can be analyzed using the U–Pb method. **(B)** Suevite, a polymict impact breccia with dark, elongated *flädle* of impact glass from the Ries crater, Germany (Katzenstein Castle near Dischingen, Baden-Württemberg). Impact glass is commonly used as sample material for Ar–Ar geochronology. **(C)** A green, glassy Ries tektite (moldavite) found in Besednice, Czech Republic. **(D)** Concordia (Wetherill) diagram showing U–Pb geochronologic results for zircon in impact melt rock from the Rochechouart impact structure in France (unpublished data). **(E)** Shocked zircon grain with LA-ICP-MS laser ablation pit created during U–Pb analysis in impact melt rock from the Charlevoix impact structure, Québec, Canada (backscattered electron image) (Schmieder *et al.*, 2019). **(F)** Argon–argon age diagram showing a well-defined plateau age, including relevant statistics for a Ries tektite sample similar to the one shown in **(C)** (from Schmieder *et al.*, 2018a). LA-ICP-MS, laser ablation inductively coupled plasma mass spectrometry.

Both the Wells Creek and Lockne impact craters described above have no or little recognized impact melt, respectively, that could potentially be used as material for radioisotopic analysis. However, a relatively large number of terrestrial impact structures have preserved impact melt-bearing rocks (*e.g*., Dence, 1971; von Engelhardt, 1984; Dressler and Reimold, 2001; Stöffler and Grieve, 2007; Osinski *et al.*, 2018), such as the up to ∼2.5 km-thick, differentiated crystalline melt sheet (the Sudbury Igneous Complex) overlain by ∼1.5 km of melt-bearing impact breccia (the Onaping Formation) at the ∼200 to 250 km-diameter Sudbury Basin in Ontario, Canada (*e.g*., Grieve, 2006; Davis, 2008; Rousell and Brown, 2009; Grieve *et al.*, 2010); the up to ∼1.2 km-thick melt sheet at the 100 km-diameter Manicouagan impact structure in Québec, Canada (*e.g*., Floran *et al.*, 1978; Grieve, 2006; Spray *et al.*, 2010) (Fig. 3A); and the up to ∼250 m-thick melt-bearing impact breccia (suevite) of the 25 km-diameter Nördlinger Ries crater in Germany (*e.g*., von Engelhardt *et al.*, 1995; von Engelhardt, 1997; Stöffler *et al.*, 2013) (Fig. 3B). The Ries impact also produced green glassy tektites (moldavites) (Fig. 3C), distal melt ejecta found ∼200 to 500 km northeast of the crater (*e.g*., Stöffler *et al.*, 2002; Trnka and Houzar, 2002). Because of the (partial to complete) resetting of geochronometers, for example, the U–Pb and K–Ar system, during high-temperature melting and degassing (diffusion) events such as major impacts (*e.g*., Jourdan *et al.*, 2012), impact melt lithologies are in most cases suitable for geochronologic analysis using a variety of radioisotopic geochronometers.

#### 2.2.1. U–Pb ages

One method used to determine impact ages is the uranium–lead (U–Pb) and coupled lead–lead (Pb–Pb) geochronometer (*e.g*., Nier, 1939; Wetherill, 1956, 1963; Tera and Wasserburg, 1972, 1974; and see Corfu, 2013 and Schoene, 2014 for reviews of its historical development and application). The U–Pb geochronometer is today used with several different technical setups. These include laser ablation inductively coupled plasma mass spectrometry (LA-ICP-MS), SIMS (SIMS and nanoSIMS), sensitive high-resolution ion microprobe (SHRIMP) analysis, and thermal ionization mass spectrometry after chemically abrading the mineral sample for better results (CA-TIMS). The latter, again, comes in different variations (isotope dilution, ID-TIMS; and total evaporation, TE-TIMS) (*e.g*., Davis, 2008). Each of these techniques has its advantage and disadvantage. While LA-ICP-MS and SIMS/SHRIMP are routinely and rapidly applied to thin-section or grain mount samples that can preserve the textural context of the sample, producing moderately precise U–Pb and Pb–Pb ages, CA-TIMS completely dissolves the mineral sample but produces much more precise ages with errors commonly in the range of a few thousands to tens of thousands of years (*e.g*., Schoene, 2014; Schaltegger *et al.*, 2015). The U-bearing minerals most commonly used for the U–Pb geochronologic analysis of impact materials are either intensely shocked or melt-grown zircon crystals (Fig. 3D, E) (*e.g*., Davis, 2008; Crow *et al.*, 2017; Kenny *et al.*, 2019a, 2019b), baddeleyite (Krogh *et al.*, 1984; Corfu and Lightfoot, 1996), monazite (*e.g*., Tohver *et al.*, 2012; Erickson *et al.*, 2017, 2019a, 2019b), and to a lesser degree titanite (Ames *et al.*, 1998) and apatite, although recent results for terrestrial impact craters suggest the latter is a promising target mineral for future studies (McGregor *et al.*, 2018, 2019). Uranium–lead results are typically visualized in a concordia diagram (Wetherill or Tera-Wasserburg plot) alongside their internal statistics (mean square weighted deviation [MSWD] and probability *p* as a measure of statistical fit) and can be corrected for a nonradiogenic (“common”) lead component. Zircon crystals from the less severely shocked, unmelted portion of the target rock of an impact structure commonly tend to yield older dates on or near concordia (the curve along which U–Pb ages from different U decay series are equal), reflecting the crystallization and/or metamorphic age(s) of the host rock (*e.g*., Schärer and Deutsch, 1990; Wielicki *et al.*, 2012; Schmieder *et al.*, 2015b). In contrast, intensely shocked and recrystallized zircon grains (so-called granular zircon, locally with μm-sized baddeleyite domains as a thermal decomposition product of zircon) (Wittmann *et al.*, 2006; Timms *et al.*, 2017) are chronometrically reset and commonly yield younger concordia ages, potentially reflecting the impact (Fig. 3D) (*e.g*., Hodych and Dunning, 1992; Krogh *et al.*, 1993; Wielicki *et al.*, 2012; Kenny *et al.*, 2019b). If the isotopic system is affected by variable loss of Pb, a discordant array of dates may define a lower intercept with concordia from which the age of the impact can be derived (*e.g*., Kamo *et al.*, 1996; Mänttäri and Koivisto, 2001). However, episodic and/or modern postimpact Pb loss can cause significant disturbance of the U–Th–Pb system, and some zircon U–Pb ages obtained for impact events (*e.g*., the Ediacaran Acraman impact in South Australia) and their geologic significance are not straightforward to interpret (Schmieder *et al.*, 2015b). A special type of zircon is typically U- and Th-rich metamict (internally radiation-damaged, pseudoamorphous) zircon (*e.g*., Pidgeon *et al.*, 1966; Meldrum *et al.*, 1998; Nasdala *et al.*, 2001), which is more susceptible to U–Pb-chronometric resetting during impact events (and other thermometamorphic processes) than nonmetamict zircon (*e.g*., Schwarz *et al.*, 2016a and unpublished data; Stockli *et al.*, 2018; McGregor *et al.*, 2019; Schmieder *et al.*, 2019). The use of metamict (domains in) shocked zircon grains in impact geochronology, therefore, warrants additional future research.

Uranium–lead and Pb–Pb ages of 3470 ± 2 Ma for zircon crystals extracted from the Paleoarchean S1 impact spherule layer in the Onverwacht Group of the Barberton Greenstone Belt in South Africa and the Warrawoona Group of the Pilbara Block in Western Australia define the oldest impact ages on Earth (Byerly *et al.*, 2002); a number of additional younger Archean and Proterozoic spherule layers occur in those regions and elsewhere (*e.g*., Glass and Simonson, 2012). Earth's oldest partially preserved impact structure is the roughly 50 km-diameter Yarrabubba impact structure in Western Australia, with a Pb–Pb age for shock-recrystallized monazite of 2229 ± 5 Ma (Erickson *et al.*, 2019a, 2019b). Shocked zircon crystals in melt rock from the ∼250 to 300 km-diameter Vredefort impact structure, the largest one and also among the three oldest on Earth, yielded a U–Pb age of 2023 ± 4 Ma (Kamo *et al.*, 1996). Zircon grains crystallized from Sudbury's impact melt sheet produced a U–Pb age of 1850 ± 1 Ma (Krogh *et al.*, 1984). In a recent study, intensely shock-metamorphosed zircon grains recrystallized into microgranular aggregates yielded a precise concordia age of 77.85 ± 0.78 Ma for the 23-km Lappajärvi impact crater in Finland (Kenny *et al.*, 2019b). This result for Lappajärvi has wider biological and astrobiological implications with respect to the role particularly of medium-sized (approximately 20–30 km-diameter) impact craters as habitats for microbial life on the early Earth (*e.g*., Kring, 2000, 2003; Osinski, 2003, 2011; Osinski *et al.*, 2001; Cockell *et al.*, 2003; Cockell, 2006) and, possibly, Mars (*e.g*., Newsom, 1980; Rathbun and Squyres, 2002; Abramov and Kring, 2005; Rummel *et al.*, 2014; Osinski *et al.*, 2017) (see also discussion in Section 4).

#### 2.2.2. Ar–Ar ages

Another technique prominently used in impact geochronology is the ^40^Ar/^39^Ar (henceforth simply Ar–Ar) method pioneered by Wänke and König (1959) and Merrihue and Turner (1966), an improved variation of the classical K–Ar technique. McDougall and Harrison (1999) and Kelley (2002) provide useful and comprehensive reviews. After sample selection, processing, and meticulous handpicking of virtually fresh and clast-poor sample splits (typically particles of impact melt rock, ideally separated into the melt groundmass and clast portion therein; impact glass; or feldspar ≤500 μm in particle size) (*e.g*., Schmieder and Jourdan, 2013a; Swindle *et al.*, 2014), the potassium-bearing rock or mineral sample, together with standard minerals, is first irradiated by fast neutrons to produce ^39^Ar from ^39^K as a proxy for K in the sample; the argon isotope ratios in the aliquots are then measured in a mass spectrometer (*i.e*., thereby eliminating the need to determine a less precise ratio of absolute K and Ar concentrations from separate sample splits) and ages can be calculated by using the latest K decay constants and standard mineral ages (Renne *et al.*, 2010, 2011). The Ar–Ar method is today most commonly applied by using the total fusion of a sample with a laser (*e.g*., Kelley and Spray, 1997) or, alternatively, the stepwise heating of a sample using a resistance furnace or laser (*e.g*., Bottomley *et al.*, 1990; Swisher *et al.*, 1992; Jourdan, 2012; Schmieder *et al.*, 2018a). Generally, the step-heating method produces a more comprehensive set of data than the total-fusion method and allows for a more robust statistical assessment of resulting ages (*e.g*., Jourdan *et al.*, 2008, 2011; Schmieder and Jourdan, 2013a).

Argon–argon results for impact structures can be disturbed by the effects of sample alteration causing the diffusive loss of radiogenic ^40^Ar* and younger apparent ages (*e.g*., Schmieder *et al.*, 2014a), and also the incorporation of inherited ^40^Ar* with inclusions of incompletely degassed older target rock material and/or excess argon from Ar-bearing fluids interacting with the sample, both causing older apparent ages (inherited and excess argon are summarized under the term “extraneous argon”) (*e.g*., Kelley, 2002). Such effects can be identified, quantified, and corrected for using the isochron approach (*e.g*., Roddick, 1978; Kuiper, 2002; Jourdan *et al.*, 2008, 2011; Jourdan, 2012; Schmieder *et al.*, 2015a). Statistically robust Ar–Ar results ideally form a “plateau” in the age spectrum (Fig. 3F), a sequence of individual degassing steps with increasing temperature that all overlap within a narrow error limit and include most (ideally at least 70%) of the ^39^Ar extracted from the sample (*e.g*., Jourdan, 2012). They are, moreover, characterized by their internal statistics expressed through MSWD and *p* values for plateau sections and isochrons (and are typically reported with 2σ errors; that is, at the ∼95% confidence level, as is done in this work unless otherwise stated). Precise Ar–Ar ages have been obtained for a number of impacts on Earth, such as 66.052 ± 0.031 Ma for glassy microtektites from the 180 km-diameter Chicxulub crater linked to the end-Cretaceous mass extinction (Renne *et al.*, 2018). High-precision Ar–Ar results for the Chicxulub microtektites at the K/T boundary (more recently also known as the K/Pg boundary) were recently used to calibrate the timing and duration of the contemporaneous reverse magnetic chron C29r (Sprain, 2017; Sprain *et al.*, 2018). Similarly, Ries tektites (Fig. 3C) yielded a precise Ar–Ar age of 14.808 ± 0.038 Ma (Schmieder *et al.*, 2018a) that can also be used to (re-)calibrate the paleomagnetic and orbitally tuned astronomical timescale (Schmieder *et al.*, 2018b). An increasingly robust intercalibration between the U–Pb and Ar–Ar geochronometers (*e.g*., Villeneuve *et al.*, 2000; Ramezani *et al.*, 2005; Renne *et al.*, 2010, 2011) provides growing confidence that ages obtained when using both techniques are not only precise (*i.e*., with a small error) but also accurate (*i.e*., close to the “true” age) and can be directly compared and correlated.

As the K decay constants and ages for standard (monitor) minerals in ^40^Ar/^39^Ar geochronology have been continuously refined (*e.g*., Steiger and Jäger, 1977; Renne *et al.*, 2010, 2011), modern Ar–Ar ages are today directly comparable with U–Pb ages (*e.g*., Renne *et al.*, 2013, 2018; Sprain *et al.*, 2015, 2018; Clyde *et al.*, 2016). This, however, also means that “legacy” Ar–Ar ages published in the older literature are, in many cases, inaccurate and require recalculation (*e.g*., Jourdan *et al.*, 2012; Schwarz *et al.*, 2015; Mercer and Hodges, 2016; Schmieder *et al.*, 2018a). Table 1 contains the most current Ar–Ar ages that were (re-)calculated, where possible, using the revised K decay constants and monitor ages of Renne *et al.* (2010, 2011). For example, the original melt rock age of 64.98 ± 0.05 Ma (1σ) for the Chicxulub impact crater, Mexico, published by Swisher *et al.* (1992) using the K decay constants of Steiger and Jäger (1977) and the Fish Canyon sanidine (FCs) standard with a then-reported age of 27.84 Ma, becomes 66.05 ± 0.18 Ma (2σ) after the recalculation of individual step ages, plateau sections and ages, and weighted mean (average) ages (*n* = 3 plateau ages; MSWD = 0.18; *p* = 0.84) obtained from those results using Isoplot 4.15 (Ludwig, 2008) and the ArAR tool of Mercer and Hodges (2016). This recalculated age is within uncertainty indistinguishable from the more recent U–Pb age of 66.021 ± 0.081 Ma for zircon crystals in ash layers around the K/T boundary in the Denver Basin (Clyde *et al.*, 2016). It is also equivalent to Ar–Ar results of 66.038 ± 0.049 Ma for glassy microtektites found at the K/T boundary in Beloc, Haiti (Renne *et al.*, 2013; Sprain *et al.*, 2015), an age of 66.051 ± 0.031 Ma for similar microtektites at a K/T section exposed on Gorgonilla Island off the coast of Colombia (Renne *et al.*, 2018), and an age of 66.052 ± 0.043 Ma for tephra in the “Iridium Z coal” layer ∼1 cm above the iridium anomaly of the K/T boundary interval (Renne *et al.*, 2013; Sprain *et al.*, 2018). The ∼24 km-diameter Boltysh impact structure in Ukraine, another end-Cretaceous impact structure (Kelley and Gurov, 2002), has a recalculated age of 65.80 ± 0.67 Ma that is, within a somewhat larger error envelope, identical to the age of the Chicxulub impact (Jourdan, 2012). However, from the identification of distal Chicxulub ejecta in the basal lake sediments of the Boltysh crater, we know that this impact predates Chicxulub by a few thousand years (Jolley *et al.*, 2010).

Likewise, through recalculation, the age of the ∼35 km-diameter Manson impact structure, Iowa (decades ago still a contender for the K/T boundary impact site), also sees a notable shift from 74.1 ± 0.1 Ma (Izett *et al.*, 1998) to an older recommended age of 75.9 ± 0.1 Ma (Table 1). The ∼100 km-diameter Popigai impact structure in Russia, with a previously recommended Ar–Ar age of 35.7 ± 0.2 Ma (Bottomley *et al.*, 1997) has, after a reinterpretation of the original Ar–Ar results, a more conservative recalculated age of 36.63 ± 0.92 Ma, which accounts for the spread of ∼1 Myr between four plateau ages, not all of which overlap (*n* = 4 plateau ages; MSWD = 7.6; *p* = 0.000) (see also Jourdan *et al.*, 2009). From this recalculation, a time gap of at least ∼0.5 Myr (and up to ∼3 Myr) seems to occur between Popigai and the somewhat younger (34.86 ± 0.32 Ma) ∼40 to 45 km-diameter Chesapeake impact structure (*a.k.a.* Chesapeake Bay; final collapsed diameter ∼85 km) on the East coast of the United States (Assis Fernandes *et al.*, 2019). This asteroid “one-two punch” is in agreement with the occurrence of two relatively closely spaced, but separate, distal ejecta layers in the Upper Eocene (Glass *et al.*, 1985; Koeberl, 2009) (Table 2), known as the older clinopyroxene layer geochemically linked to the Popigai impact (Whitehead *et al.*, 2000) and the younger North American (micro-)tektites linked to the Chesapeake impact (Deutsch and Koeberl, 2006).

In a few cases, recalculation of the original Ar–Ar results was omitted due to potentially unreliable standard ages used in the original geochronologic analysis. This, for example, applied to ages obtained using the B4M muscovite standard, which was commonly used in geochronology laboratories in the 1980s (*e.g*., for the Haughton impact structure, Canada) (Jessberger, 1988) and later (for the Ilyinets impact structure, Ukraine) (Pesonen *et al.*, 2004). The B4M standard was recently shown to be quite heterogeneous in composition and age between finer- and corser-grained domains of the muscovite and is, therefore, not recommended as a standard in modern Ar–Ar geochronology (Heri *et al.*, 2014). Finally, some impact structures, predominantly those in Russia and Ukraine, have only K–Ar ages (*e.g*., Val'ter *et al.*, 1981; Gurov *et al.*, 2009). Because any possible disturbance of the isotopic system (*e.g*., alteration or contamination with older material as outlined above) cannot be identified and quantified, K–Ar age values should be treated as “ballpark” numbers until more robust Ar–Ar results are available.

### 2.3. Rb–Sr, (U–Th)/He, and ^14^C ages

The Rb–Sr method has been applied to impact melt lithologies and mineral separates from a number of terrestrial impact structures (*e.g*., Reimold *et al.*, 1981; Deutsch *et al.*, 1992). However, Rb–Sr ages are notoriously unreliable due to the high mobility of Rb and Sr and, consequently, the susceptibility of the Rb–Sr isotopic system to alteration (*e.g*., Jourdan *et al.*, 2009; Nebel *et al.*, 2011; Schmieder *et al.*, 2015a). Today, all of the older Rb–Sr ages for terrestrial impact structures (*e.g*., Reimold *et al.*, 1981) have been superseded by more robust U–Pb and/or Ar–Ar ages and, therefore, none of the original Rb–Sr results is recommended as best-estimate ages in this summary (Table 1).

The low-temperature (U–Th)/He geothermochronometer can monitor the cooling of impact lithologies to temperatures below approximately 200–180°C using zircon and ∼110–40°C using the less He-retentive mineral apatite (*e.g*., Stockli *et al.*, 2000; Farley and Stockli, 2002; Farley, 2002; Reiners *et al.*, 2004; Reiners, 2005). While (U–Th)/He analyses of uplifted basement rocks at the large Manicouagan impact structure resulted in ages younger than the impact age due to slow cooling and postimpact He loss (van Soest *et al.*, 2011; Biren *et al.*, 2014), (U–Th)/He age determinations for other terrestrial impact structures and distal ejecta deposits yielded ages that are, within error, consistent with U–Pb and Ar–Ar ages (Young *et al.*, 2013; Biren *et al.*, 2019) and precise stratigraphic ages (Wartho *et al.*, 2012). In the absence of more robust stratigraphic and isotopic age constraints, a (U–Th)/He age of 663 ± 90 ka currently represents the most reasonable estimate for the age of the ∼350 m-diameter Monturaqui impact crater in the Chilean Andes (Ukstins Peate *et al.*, 2010).

Finally, the ^14^C (radiocarbon) method has occasionally been applied to charcoal and other types of organic material found at geologically young impact craters, such as the Xiuyan crater in China (>50 ka) (Liu *et al.*, 2013) and the Kaali and Ilumetsa impact crater fields in Estonia (Losiak *et al.*, 2016, 2017, 2019). Because of the short half-life of ^14^C of ∼5730 years, the method fails to determine ages older than roughly 50,000 years (Hughen *et al.*, 2004; Muscheler *et al.*, 2014).

### 2.4. Other ages

Impact ages obtained via different methods, such as fission track analysis (on zircon, apatite, or glass) (*e.g*., Bigazzi and De Michele, 1996), cosmogenic nuclides and exposure ages (*e.g*., Marrero *et al.*, 2010; Barrows *et al.*, 2019), luminescence (*e.g*., Prescott *et al.*, 2004), or paleomagnetic methods (*e.g*., Pesonen *et al.*, 1999; Lepaulard *et al.*, 2019), were selected as best-estimate ages, provided they agree with the local geologic constraints. Recent reviews of fission track analysis and its application in the Earth sciences are provided by Malusà and Fitzgerald (2019) and articles therein. This technique, based on the identification of damage trails in crystals and glasses induced by the spontaneous fission of ^238^U in the sample and their density (*e.g*., Kohn *et al.*, 2019), has been applied to impact lithologies ever since their discovery (*e.g*., Gentner *et al.*, 1967, 1969; Koeberl *et al.*, 1993; McHone and Sorkhabi, 1994; Weber *et al.*, 2005). In the case of the 1.13 km-diameter Tswaing impact crater in South Africa, a fission track age of 220 ± 104 ka for impact glass (Storzer *et al.*, 1999) is preferred over a very poorly constrained stratigraphic age (<2.05 Ga) and Ar–Ar results that are disturbed toward more ancient apparent ages due to the presence of inherited ^40^Ar* sourced from the Paleoproterozoic granitic target rock (Jourdan *et al.*, 2007). Sometimes, these geochronologic techniques provide the only age constraints for an impact structure other than the (maximum) stratigraphic age.

## 3. Result: A List of Recommended Ages for Terrestrial Impact Structures and Deposits

Significant work on the terrestrial impact structures has produced a large number of ages of different type and quality (*e.g*., for the Nördlinger Ries in Germany) (Buchner *et al.*, 2010a, 2010b, 2013 and references therein; Schmieder *et al.*, 2018a, 2018b). In such cases, the most precise and accurate ages obtained by using modern isotopic techniques, in line with geologic and stratigraphic constraints, were carefully chosen as the recommended best-estimate impact age. Stratigraphic constraints were calibrated using the latest International Chronostratigraphic Chart (ICS; updated, v2018/08) (Cohen *et al.*, 2013). It is important to keep in mind that stratigraphic ages in the ICS may (slightly) change in the future as those ages are refined.

**FIG. 4.**
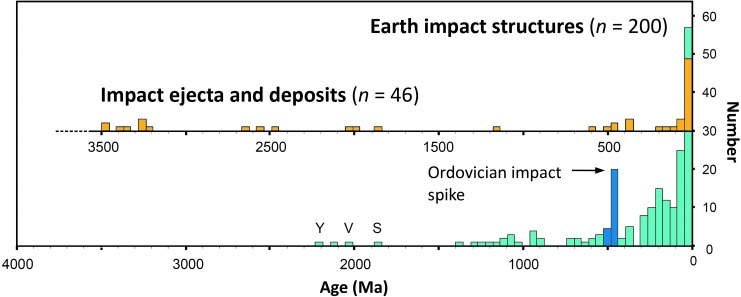
Histogram showing the age distribution of terrestrial impact structures (blue) and ejecta deposits (orange). Ejecta layers that presumably have the same age and occur at more than one locality (*e.g*., the ∼3470 Ma Paleoarchean S1 Barberton and Warrawoona spherule layer identified in South Africa and Western Australia, respectively) are shown as one deposit. Ages are average ages (*e.g*., 2100 ± 400 Ma for Dhala, India, shows as an age at 2100 Ma). Note the distinct Ordovician impact spike around ∼470 to 450 Ma (darker blue). Note this diagram does not distinguish between larger and smaller impacts. S, Sudbury (Krogh *et al.*, 1984; Davis, 2008); V, Vredefort (Kamo *et al.*, 1996); Y, Yarrabubba (Erickson *et al.*, 2019a, 2019b). Compare Table 1 with ages for impact structures and Table 2 with ages for ejecta deposits.

The recommended terrestrial impact ages (*n* = 200) are listed in Table 1, and ages for impact deposits (distal and proximal ejecta; *n* = 46) are listed in Table 2. Both tables are sorted by age, with the youngest impact structures and deposits on top and the oldest ones at the bottom. Twenty impact structures have either stratigraphic or isotopic ages with relative errors ≤1% (*e.g*., Chicxulub and the Ries); 36 have errors ≤2%. All terrestrial impact ages are, in addition, plotted in histograms in Fig. 4. They can be used to:

(1) reconstruct and quantify the impact (mass) flux in the inner Solar System and, in particular, the Earth–Moon system through geologic time, thereby assessing Earth's impact rate (*e.g*., Grieve and Dence, 1979; Montanari *et al.*, 1998; Grieve, 2001a, 2001b; Bland, 2005; Meier and Holm-Alwmark, 2017; Mazrouei *et al.*, 2019);
(2) utilize impact ejecta as event markers in the (bio-)stratigraphic record and to refine magneto-stratigraphy, for example, around the K/T boundary (*e.g*., Sprain *et al.*, 2015, 2018) and in the Neogene stratigraphic record (*e.g*., Schmieder *et al.*, 2018a, 2018b);
(3) test models of synchronous double or multiple impacts in the terrestrial record, such as that proposed for the apparent East and West Clearwater Lake impact crater doublet in Québec, Canada (*e.g*., Dence *et al.*, 1965; cf. Schmieder *et al.*, 2015a), and the postulated Late Triassic terrestrial impact crater chain (*e.g*., Spray *et al.*, 1998; cf. Schmieder *et al.*, 2010b, 2014a);
(4) assess the potential link between large impacts and mass extinction and diversification events in the biosphere, exemplified most dramatically by the Chicxulub impact at the K/T boundary (*e.g*., Alvarez *et al.*, 1980; Rampino, 1999; Grey *et al.*, 2003; Schmitz *et al.*, 2008; Schulte *et al.*, 2010; Racki, 2012; DePalma *et al.*, 2019);
and
(5) constrain the duration of melt sheet crystallization in large impact craters (*e.g*., Davis, 2008; Kenny *et al.*, 2019a) and the lifetime of hydrothermal systems in cooling impact craters (*e.g*., Ames *et al.*, 1998; Abramov and Kring, 2004, 2007; Schmieder and Jourdan, 2013a, 2013b; Pickersgill *et al.*, 2019a; Kenny *et al.*, 2019b), which may have served as potential habitats for microbial life on the early Earth and possibly also Mars (*e.g*., Kring, 2000; Rathbun and Squyres, 2002; Cockell *et al.*, 2003; Osinski *et al.*, 2013; Rummel *et al.*, 2014).

## 4. Discussion

### 4.1. Considerations on the terrestrial impact flux from the age distribution

With a representative set of precise and accurate isotopic ages for terrestrial impacts, as well as stratigraphic ages within their generally larger envelope of uncertainty (Tables 1 and 2), one can examine and re-evaluate the potential temporal connection between impact events on Earth themselves and the overall terrestrial impact cratering record (*e.g*., Grieve and Dence, 1979; Grieve and Robertson, 1979; Grieve, 1987, 1991, 2001a, 2001b; Grieve and Pesonen, 1996).

As more impact structures are discovered and their ages determined and refined, a population of the Phanerozoic impact structures and deposits stands out: those with Ordovician ages. The Ordovician period spans the time between ∼485 and ∼443 Ma (Cohen *et al.*, 2013). At present, 22 of the currently known 200 impact structures on Earth, that is, more than 10%, have proven or very likely Ordovician ages, creating a distinct age spike in the terrestrial impact cratering record. A representative histogram is shown in Fig. 4. Recent additions to the list of (very likely) Ordovician impacts, based on new U–Pb and Ar–Ar geochronologic results, include, for example, the 54 km-diameter Charlevoix impact structure (453–430 Ma via LA–ICP–MS U–Pb on zircon in impact melt rock) (Schmieder *et al.*, 2019), the 50 km-diameter Carswell impact structure (Alwmark *et al.*, 2017), and the 8 km-diameter La Moinerie impact structure (McGregor *et al.*, 2019), all three located in Canada; as well as the 18 km-diameter Lawn Hill impact structure in Australia (Darlington *et al.*, 2016). Those impact structures, six in the United States, nine in Canada, five in Sweden, and one in Estonia, Ukraine, and Australia, respectively, were produced over several million years (*e.g*., Grahn *et al.*, 1996; Alwmark *et al.*, 2010). In addition, a large number of fossil meteorites found in Ordovician limestone in Sweden (*e.g*., Schmitz *et al.*, 1996, 2001) and the impact-produced Osmussaar Breccia in Estonia (Alwmark *et al.*, 2010) testify to a period of enhanced bombardment of Earth by asteroids at that time. Analysis of the fossil meteorites and impact breccias suggests that most of the Ordovician impacts are linked to the collisional breakup of the L-chondrite parent asteroid in space some 470 Myr ago (*e.g*., Ar–Ar results of Bogard *et al.*, 1976, 1995; Korochantseva *et al.*, 2007; Swindle *et al.*, 2014), which then sent large masses of partially shock-melted stony meteorites into Earth-crossing orbits. Extraterrestrial chromite grains extracted from resurge deposits of the Lockne impact structure and the Osmussaar Breccia indicated an L-chondritic source (Alwmark and Schmitz, 2007; Alwmark *et al.*, 2010). Geochemical analysis of impact melt rock from the East Clearwater Lake impact structure in Canada also suggested an ordinary (possibly L-) chondritic impactor (Palme *et al.*, 1979; McDonald, 2002; Daly *et al.*, 2018). However, the Ordovician bombardment of Earth was one of numerous but predominantly relatively small asteroids.

Apparent “clusters” of impacts, that is, two or more impact events with overlapping or nearly overlapping ages, also seem to occur in geologic times other than the Ordovician. For example, at least four impact structures, Popigai in Russia (Bottomley *et al.*, 1997, Ar–Ar age recalculated), Chesapeake in the United States (Assis Fernandes *et al.*, 2019), and Wanapitei (Bottomley *et al.*, 1979, recalculated) and Mistastin in Canada (Sylvester *et al.*, 2013), have isotopic ages that all fall in the time range between ∼38 and ∼35 Ma in the Late Eocene (Cohen *et al.*, 2013). However, not all of their (recalculated) ages overlap (*n* = 4 impact crater ages; MSWD = 114; *p* = 0.000). From the age distribution (and the associated uncertainty) alone, the formation of four larger impact structures within a few million years may appear like the usual background production when considering the effective impact crater distribution and cratering rate (Wanapitei-sized impact craters are statistically produced every ∼60,000 years; Mistastin-sized craters every ∼600,000 years; Chesapeake-sized craters every ∼4.5 Myr; and Popigai-sized impact craters every ∼26 Myr) (*e.g*., Grieve and Shoemaker, 1994; French, 1998). However, a distinct ∼2.5 Myr-long spike in extraterrestrial ^3^He in pelagic limestone (Farley *et al.*, 1998), in combination with a strong enrichment in extraterrestrial chromite grains in Upper Eocene sediments of the Global Boundary Stratotype Section and Point (GSSP) for the Eocene–Oligocene at Massignano, Italy, (Schmitz *et al.*, 2015; Boschi *et al.*, 2017), argues for a Late Eocene asteroid (or comet) shower, thereby potentially producing a distinct impact cluster. One mechanism that can explain the formation of clusters in the terrestrial impact crater record is one or more impacts in space causing the breakup of large asteroids into families of asteroids, members of which can then be delivered to the Earth (*e.g*., Zappalà *et al.*, 1998; Nesvorný *et al.*, 2002, 2006; Farley *et al.*, 2006; Bottke *et al.*, 2007; Claeys and Goderis, 2007; Schmitz *et al.*, 2008). Trace element analysis of impactites suggested that the Popigai and Wanapitei impact structures both had L-chondritic impactors (Masaitis and Raikhlin, 1986; Tagle and Claeys, 2004, 2005; Tagle and Hecht, 2006; Tagle *et al.*, 2006), although Kyte *et al.* (2011) argued that the Popigai-derived Upper Eocene clinopyroxene spherule layer may be linked to the impact of an H-chondrite. The nature of the impactor that produced the Chesapeake crater is, at this point, still somewhat uncertain (McDonald *et al.*, 2009; Goderis *et al.*, 2010). The geochemical and oxygen isotopic analysis of extraterrestrial chromite grains found in Upper Eocene sediments at Massignano indicates an H-chondritic source for the Popigai impact and an L-chondritic source for the somewhat younger Chesapeake impact (Schmitz *et al.*, 2015; Boschi *et al*., 2017).

In addition to seemingly clustered impacts, the recognition of an apparent periodic pattern in the timing of impact events has caused a debate that started in the mid-1980s and still continues today. Following Raup and Sepkoski (1984), who found that mass extinctions in the Phanerozoic seem to have a periodic pattern potentially caused by extraterrestrial forces (such as periodic cometary showers), other researchers also recognized through time-series analysis that large impacts occurred in a similar repetitive pattern of predominantly ∼26 and ∼30 Myr intervals over the past ∼250 Myr and may, therefore, be causally linked (*e.g*., Alvarez and Muller, 1984; Davis *et al.*, 1984; Rampino and Stothers, 1984; Torbett and Smoluchowski, 1984; Muller, 1985; Rampino and Haggerty, 1996; Rampino and Caldeira, 2015, 2017). However, one should keep in mind that those periodicity models were based on the impact crater ages available in the 1980s and 90s, and since then, other workers have called the proposed periodicity into question (*e.g*., Grieve *et al.*, 1988; Heisler and Tremaine, 1989; Baksi, 1990; Weissman, 1990; Yabushita, 1996; MacLeod, 1998; Montanari *et al.*, 1998; Bailer-Jones, 2011), some of them noting that the apparent periodicity may, in part, be an artificial effect due to the rounding of imprecise impact ages to integer values, often in multiples of 5 or 10 Ma (*e.g*., Jetsu and Pelt, 2000; Grieve and Kring, 2007). More recently, Meier and Holm-Alwmark (2017) demonstrated that the apparent periodic pattern in Earth's impact events, at least those filtered for reasonably precise and accurate age constraints (compare Baksi, 1990), may be related to clusters of impacts with similar ages that seem to be the main carriers of the periodic signal. Based on refined statistics, they argued that there is currently no evidence for periodicity in the terrestrial impact record when up-to-date impact crater ages are used as input parameters. Ages presented in Tables 1 and 2 of this work aim to help resolve such issues and debates.

In the context of seemingly periodic impacts and extinction events (Raup and Sepkoski, 1984) and the “kill curve” of Raup (1990), we also refer to the role of impacts in Earth's biosphere (Section 4.4).

**FIG. 5.**
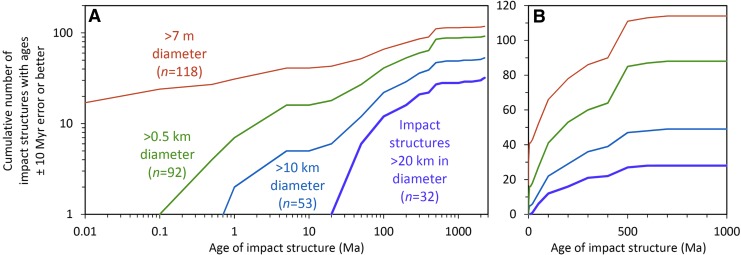
Cumulative number of impact structures with more or less well-established ages (±10 Ma in error) versus time for the entire Earth and including different crater size populations (compare, e.g., Grieve, 1984; Mazrouei *et al.*, 2019). **(A)** Log–log plot over >2 Gyr; **(B)** linear plot for the past 1 Gyr [same color scheme as in **(A)**].

Precise and accurate impact ages, moreover, help constrain the preserved terrestrial crater size–frequency distribution and, by inference, estimate the impact cratering rate in the Earth–Moon system in the past. Figure 5 shows the cumulative number of Earth's impact structures of variable size with reasonably well-constrained ages (±10 Ma error) for the entire Earth, including very small impact craters (and pits) ∼7 to 500 m in diameter (which are usually not plotted because they are preferentially removed from Earth's record by erosion; *e.g*., Grieve and Dence, 1979; Hughes, 2000). Because the terrestrial impact record is incomplete for several reasons outlined earlier (*e.g*., Johnson and Bowling, 2014; Hergarten and Kenkmann, 2015) (Fig. 1), the lunar impact record and its crater size–frequency distribution are commonly used as a proxy for the impact crater production rate on Earth (*e.g*., Neukum and Ivanov, 1994; Neukum *et al.*, 2001; Werner *et al.*, 2002; Ivanov *et al.*, 2003). Additional constraints come from the size–frequency distribution of near-Earth asteroids (*e.g*., Shoemaker *et al.*, 1979; Durda *et al.*, 1998; Morbidelli, 1999; Bottke *et al.*, 2000; Werner *et al.*, 2002; Stuart and Binzel, 2004; Michel and Morbidelli, 2007; Le Feuvre and Wieczorek, 2011; Johnson and Bowling, 2014; Wheeler and Mathias, 2019), the population of Earth-crossing comets, the Sun's position in the galactic plane (e.g., Shoemaker, 1998b; Ye, 2018), as well as the distribution of extraterrestrial ^3^He (Farley, 1995, 1998, 2001), platinum-group metals (Peucker-Ehrenbrink, 2001), and fossil meteorites and extraterrestrial chromite grains (*e.g*., Schmitz *et al.*, 1996, 2001, 2015; Heck *et al.*, 2004; Alwmark and Schmitz, 2009; Schmitz, 2013) in marine sediments. While some authors proposed that the impact flux in the Earth–Moon system has continuously declined over the past 3 Gyr (Minton and Malhotra, 2010), others suggested that the impact flux has remained more or less stable over the last 2 Gyr (*e.g*., Neukum and Ivanov, 1994; Hörz, 2000; Hughes, 2000). Part of this debate is whether the Earth has seen a significant increase of impacts, particularly those producing craters >20 km in diameter, over the last few hundred Myr—perhaps by a factor of two or three (*e.g*., Grieve, 1984; McEwen *et al.*, 1997; Shoemaker, 1998a, 1998b; Hughes, 2000; Bland, 2005; cf. Grier *et al.*, 2001). More recently, Mazrouei *et al.* (2019) suggested that the terrestrial impact flux experienced an increase by a factor of 2.6 some 290 Myr ago. It is beyond the scope of this geochronology-focused article to assess Earth's effective impact cratering rate, but while Bland (2005) provides a useful summary and discussion, the list of recommended impact ages (Tables 1 and 2) may help place additional constraints on the Proterozoic (2.5 Ga to ∼541 Ma) and Phanerozoic (∼541 Ma until present) terrestrial impact crater production.

### 4.2. Impact-delivered extraterrestrial mass accreted on Earth over time

While the distribution of impact ages in the geologic time line suggests that the Earth was hit by asteroids (and/or comets) more frequently during, for example, the Ordovician compared with other periods of time, it is important to note that this temporal distribution is biased by various factors. First, the terrestrial impact cratering record is, with currently 200 impact structures recognized on Earth, very limited and, therefore, not representative of a planetary production record (*e.g*., Grieve and Dence, 1979; Johnson and Bowling, 2014). Because the majority of impactors hit the seafloor (particularly during geologic times with supercontinents) and the oceanic crust has been tectonically recycled in multiple Wilson cycles over ∼2 Gyr (*e.g*., Scotese, 2001), most impact structures have been removed from Earth's surface (*e.g*., Johnson and Bowling, 2014; Hergarten and Kenkmann, 2015). With the exception of the ∼20 to 40 km-diameter Jurassic–Cretaceous Mjølnir impact structure off the coast of Norway (Dypvik *et al.*, 1996), the ∼45 km-diameter Eocene Montagnais impact structure on the Scotian Shelf of eastern Canada (Jansa *et al.*, 1989), and evidence for the Pleistocene submarine Eltanin impact (Gersonde *et al.*, 1997), no impact structures are currently known on the present-day seafloor. Second, some countries (*e.g*., the United States, Canada, Australia, and many European countries) have a longer tradition in impact crater research compared with others (*e.g*., China), which may cause an apparent preponderance of impacts in those countries and their respective geologic settings. Australia and Finland, for example, have a relatively high density of preserved Precambrian impact structures because much of their landmass consists of Archean and Proterozoic rocks that can preserve this old cratering record (Fig. 2). Third, impact ages can be more precisely determined stratigraphically in well-characterized sedimentary target settings similar to that at the Lockne crater, Sweden, than in others (*e.g*., Île Rouleau, Canada), an effect that probably contributes to the impact age spike in the Ordovician. Lastly, the impact age distribution shown in Fig. 4 does not take into account the actual magnitude of the impact events that occurred over time, which can be expressed by the mass of projectile material delivered to Earth during impact and the corresponding impact energy (half of the projectile mass times the impact velocity squared) (*e.g*., French, 1998).

**FIG. 6.**
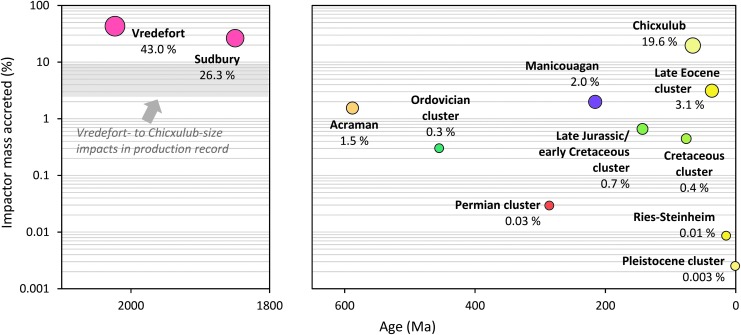
Graph showing calculated percentage of impactor mass accreted on Earth (logarithmic scale) over the past ∼2 Gyr of geologic time (linear scale) relative to the preserved impact crater record (*n* = 200) as a quantitative measure of the terrestrial impact flux (numbers calculated using equations in Abramov *et al.*, 2012 and best-estimate geologic constraints as input parameters). Note the given percentage values strongly overrepresent these individual impacts when the complete production record over ∼2 Gyr is taken into account; only ∼15% to 25% of that record is today observed on Earth. Impact crater populations apparent in age distribution diagrams (Fig. 4) may not be very prominent in this type of diagram when they consist of a large number of medium-sized and smaller craters. Apparent impact clusters: Ordovician: 22 impact structures with proven and very likely Ordovician ages; Permian: West Clearwater Lake, Terny and Douglas; Late Jurassic/Early Cretaceous: Morokweng, Mjølnir, and Dellen; Cretaceous: Kara, Manson, and Lappajärvi (∼78 to 70 Ma); Late Eocene: Popigai, Chesapeake, Mistastin, and Wanapitei (∼38 to 34 Ma); Pleistocene: Bosumtwi, Zhamanshin, and Pantasma (∼1.1 to 0.8 Ma). Color scheme as in Fig. 2 and the International Stratigraphic Chart.

An alternative and perhaps more informative way of representing the impact flux through geologic time is plotting the accreted impactor mass versus time (Fig. 6). By using equations modified after the work of Abramov *et al.* (2012) and well-established impact crater scaling laws (*e.g*., Grieve *et al.*, 1981; Lakomy, 1990; Melosh, 1989), along with reasonable constraints on the type of impactors (*e.g*., Tagle and Hecht, 2006; Goderis *et al.*, 2012; Koeberl, 2014), their bulk density (*e.g*., Consolmagno and Britt, 1998; Consolmagno *et al.*, 2008; Macke, 2010; Macke *et al.*, 2011), different types of target rock (*e.g*., Abramov *et al.*, 2012), and variable impact velocities (*e.g*., between 10 and 20 km^−1^), the absolute and relative mass flux can be calculated. However, because many of the input parameters are associated with significant uncertainties, these calculations can only provide approximate first-order estimates. For this purpose, we calculated the mass of the three largest impacting bodies based on transient crater diameter values in the literature (125 km for Vredefort, 110 km for Sudbury, and 100 km for Chicxulub) (Stöffler *et al.*, 1994; Kring, 1995, 2005; Therriault *et al.*, 1997; Grieve and Therriault, 2000). Moreover, such calculations do not take into account the mass accreted from potentially large impacts on Earth that created the Archean spherule layers because the size and type of those projectiles are not well constrained. (One could potentially use the thickness of an ejecta layer as a gauge for the corresponding impactor size, but distal ejecta layers become thinner with distance from their source crater and postimpact sedimentary reworking commonly modifies the thickness of fallout deposits; *e.g*., McGetchin *et al.*, 1973; Simonson *et al.*, 2000; Byerly *et al.*, 2002; Johnson and Melosh, 2012; Johnson *et al.*, 2016.) Therefore, estimates of the total accreted projectile mass based on the impact crater record alone are minimum estimates.

Doing the relative mass flux calculations for the partially preserved terrestrial impact crater record (*n* = 200) with the above caveat in mind (and not taking into account the [large] impacts that produced the terrestrial impact ejecta deposits), a few things become immediately apparent (Fig. 6): The giant Vredefort impact alone delivered >40% of the total projectile mass accreted among all 200 known crater-forming impacts over the last >2 Gyr, and the three largest impact structures on Earth—Vredefort, Sudbury, and Chicxulub—were created by projectiles that together make up ∼90% of that total impactor mass. The end-Cretaceous Chicxulub impact concentrates ∼70% of all extraterrestrial mass in the Phanerozoic impact crater record (*n* = 172). In contrast, other relatively large impacts (*e.g*., Acraman and Manicouagan) in the Ediacaran and Phanerozoic only contributed a small percentage of the total impactor mass. For example, the Ordovician impacts, creating a large group of medium-sized and smaller impact craters with proven and likely Ordovician ages (Fig. 4) (Section 4.1), only delivered ∼0.3% of the total impactor mass (Fig. 6) because those projectiles were, although numerous, relatively small. Seemingly sizeable impact events such as the Ries–Steinheim double impact ∼14.8 Myr ago (Stöffler *et al.*, 2002; Schmieder *et al.*, 2018a, 2018b) and the three largest Pleistocene impacts (Bosumtwi, Zhamanshin, and Pantasma, not including the enigmatic impact that created the large Australasian tektite strewn field) (*e.g*., Hartung and Koeberl, 1994; Cavosie, 2018; Rochette *et al.*, 2019), all producing impact craters >10 km in diameter, delivered asteroid masses that are statistically insignificant (∼0.01% or less). Such calculations put the production rate, relative abundance, and effective significance of large- versus medium-sized and small impacts through geologic time (*e.g*., Grieve and Dence, 1979; Grieve, 2001a, 2001b; Meier and Holm-Alwmark, 2017; Rampino and Caldeira, 2017; Mazrouei *et al.*, 2019) into a different perspective.

However, one should also keep in mind that the above relative impactor mass distribution is only relevant to the partially preserved impact crater record observable today (*n* = 200) and, therefore, draws a distorted image of the true impact crater production over time. Assuming Chicxulub-sized (∼180 km diameter) impacts occur approximately every 100–150 Myr (Grieve and Shoemaker, 1994; Neukum and Ivanov, 1994; French, 1998), the production record for the past ∼2 Gyr, at a more or less constant impact rate, would contain ∼20 Chicxulub- or Sudbury-sized impacts (producing ∼150 to 200 km-diameter craters), ∼77 Popigai-sized impacts (∼100 km), ∼450 Siljan-sized impacts (∼50 km), and ∼5780 Ries-sized impacts (∼20 km). Those >6000 impacts producing craters >20 km in diameter would have delivered several hundred million megatons of impactor material to Earth, only ∼6% of which would have been concentrated in the Vredefort projectile (Chicxulub ∼3%). The same calculations adjusted for an impact rate ∼2 to 3 times lower before ∼0.3 Ga (*e.g*., Shoemaker, 1998b; Mazrouei *et al.*, 2019) yield roughly 2300 impacts producing craters >20 km in diameter over ∼2 Gyr (Vredefort ∼10%; Chicxulub ∼5% of accreted impactor mass). The above calculations, depending on the cratering rate chosen, suggest that today's partial preservation record (*n* = 200) represents only some 15–25% of the impact craters produced over the past ∼2 Gyr. These estimates are broadly consistent with those of Johnson and Bowling (2014).

### 4.3. Geochronologic evidence for double and multiple impact events on Earth

**FIG. 7.**
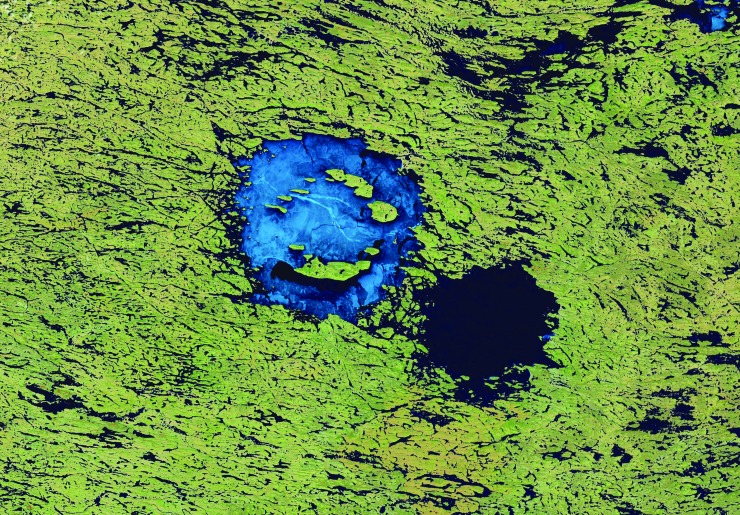
The two clearwater Lakes in Québec, Canada. The western structure, West Clearwater Lake, is ∼36 km in diameter and has a ring of islands where impact melt-bearing rocks occur. The eastern structure, East Clearwater Lake ∼26 km in diameter, has a more subtle appearance. Both impact structures were considered to represent a 290 million year-old impact crater doublet (Dence *et al.*, 1965; Reimold *et al.*, 1981) until recently. New Ar–Ar geochronologic results, however, demonstrate that the eastern crater formed during the Middle Ordovician (∼465 Ma), a time of intense asteroid bombardment of Earth, whereas the western crater formed in the Early Permian (∼286 Ma) and is therefore ∼180 Myr younger (Schmieder *et al.*, 2015a). Landsat OLI/TIRS satellite image taken on June 13, 2013, when the western lake was still partly frozen (*Source*: GloVis, USGS). Scene width ∼120 km. OLI, Operational Land Imager; TIRS, Thermal Infrared Sensor.

There has been an ongoing debate about the geologic and geochronologic evidence for double and multiple impact events on Earth (Spray *et al.*, 1998; Miljković *et al.*, 2013, 2014; Schmieder *et al.*, 2014a, 2014c, 2015a, 2016b). Classic examples of pairs of closely spaced impact craters are the ∼25 km Nördlinger Ries and ∼3.8 km Steinheim Basin in Germany (Stöffler *et al.*, 2002) and the two Clearwater Lakes in Québec, Canada (*e.g*., Dence *et al.*, 1965; Schmieder *et al.*, 2015a) (Fig. 7). While the age of the Nördlinger Ries is precisely constrained (tektite Ar–Ar age of 14.808 ± 0.038 Ma) (Schmieder *et al.*, 2018a, 2018b), the age of the Steinheim Basin is still somewhat enigmatic. However, the two impact craters are thought to be genetically linked because of their proximity, the similar age of their Middle Miocene crater lake sediments (Heizmann and Hesse, 1995), and their geometric alignment with the Central European tektite strewn field to the northeast (Stöffler *et al.*, 2002). Clearly, a representative isotopic age for Steinheim would help assess that situation with more confidence; unfortunately, previous Ar–Ar results for impact-melted sandstone and (U–Th)/He results for zircon crystals from the central uplift of the complex Steinheim impact crater failed to produce geologically meaningful results (Buchner *et al.*, 2010a).

In Canada, the larger, ∼36 km-diameter West Clearwater Lake impact structure has a ring of islands where impact melt-bearing rocks occur. East Clearwater Lake, 26 km in diameter, has a more subtle appearance and impact melt rock is only known from drillings (*e.g*., Simonds *et al.*, 1978; Reimold *et al.*, 1981; Grieve, 2006). For almost 50 years, these two impact structures had been considered a textbook example of an impact crater doublet created simultaneously by the impact of a binary asteroid (Dence *et al.*, 1965) in the early Permian some 290 Myr ago (Reimold *et al.*, 1981). However, repeated Ar–Ar analysis (Bottomley *et al.*, 1990; Schmieder *et al.*, 2015a), alongside other lines of geologic evidence (*e.g*., Scott *et al.*, 1997), eventually made a convincing case against the double impact scenario. While the larger western crater was indeed produced in the Permian at 286.2 ± 2.6 Ma (Schmieder *et al.*, 2015a), the eastern crater is almost 180 Myr older and, with an age around 465 Ma (Bottomley *et al.*, 1990; Schmieder *et al.*, 2015a; Biren *et al.*, 2016), is part of the prominent Ordovician impact crater population preserved on our planet (Fig. 4 and Table 1).

Two closely spaced impact structures similar in spatial arrangement to the Clearwater Lakes in Canada are the Suvasvesi North and South impact structures in Finland, both ∼4 km in diameter and ∼6 km apart from center to center (*e.g*., Pesonen *et al.*, 1996b; Lehtinen *et al.*, 2002). Not surprisingly, the two impact structures had previously been considered a possible crater doublet created by the impact of a binary asteroid (Werner *et al.*, 2001). However, more recent Ar–Ar and U–Pb (zircon) geochronologic results for impact melt rock samples from both structures suggest Suvasvesi South is considerably older (≥720 Ma, *i.e*., Proterozoic) than the Suvasvesi North structure (∼85 Ma, Cretaceous). Similar to the two Clearwater Lake impact structures, Suvasvesi North and South seem to constitute a “false” impact crater doublet (Schmieder *et al.*, 2014c, 2016b; Schwarz *et al.*, 2016a). In contrast, the 14 km-diameter Lockne and 0.7 km-diameter Målingen impact structures in Sweden may represent a true crater doublet (Ormö *et al.*, 2014) within the framework of multiple impacts during the Ordovician (see also Section 4.1). A review and geochronologic assessment of these and other proposed terrestrial impact crater doublets (*e.g*., Gusev and Kamensk in Russia; Movshovic *et al.* 1991; Melosh and Stansberry, 1991; Bottke and Melosh, 1996; Masaitis, 1999) are provided by Schmieder *et al.* (2014c).

While the Ordovician period can be regarded as a time of intense impact flux, there is currently no evidence for synchronous multiple impact events resulting in the formation of larger-scale impact crater chains on Earth. Although such a scenario had been proposed for at least five impact structures with overlapping ages (Manicouagan and Lake Saint Martin in Canada, Red Wing Creek in the United States, Rochechouart in France, and Obolon in Ukraine) in the Late Triassic some 214 Myr ago (Spray *et al.*, 1998), more recent Ar–Ar age determinations on the Lake Saint Martin (227.8 ± 0.9 Ma) (Schmieder *et al.*, 2014a) and Rochechouart (206.92 ± 0.32 Ma) (Cohen *et al.*, 2017; cf. Schmieder *et al.*, 2010b) impacts and refined stratigraphic age constraints for Obolon (<185 Ma) (Schmieder and Buchner, 2008) demonstrated that all of those craters have very different ages and are thus unrelated. We conclude that the Late Triassic Earth did not see a multiple impact event similar to the impact of several large fragments of comet Shoemaker-Levy 9 on Jupiter as observed by the Hubble Space Telescope in July 1994 (Crawford *et al.*, 1994). While there are geologically old impact crater chains on the Moon and other planetary bodies that formed after the impact of tidally disrupted “rubble pile” asteroids or comets (*e.g*., Wichman and Wood, 1995; Schenk *et al.*, 1996; Richardson *et al.*, 1998), no such chain is known to exist on Earth and their formation over shorter periods of geologic time is considered very unlikely (Bottke *et al.*, 1997).

### 4.4. The role of impacts and impact ages in Earth's biosphere

With the advent of the “New Catastrophism” in the wake of the impact mass extinction hypothesis, according to which Earth's Mesozoic life—most prominently the dinosaurs—was wiped out due to the impact of a large asteroid that was also the source of a global iridium anomaly (Alvarez *et al.*, 1979, 1980; Ganapathy, 1980; Hsü, 1980; Kyte *et al.*, 1980; Smit and Hertogen, 1980), larger meteorite impacts have been discussed as potential triggers for most, if not all, of the “big five” biological extinction events in the geologic past (*e.g*., Raup and Sepkoski, 1984; Raup, 1990, 1992; Hodych and Dunning, 1992; Sepkoski, 1996; Hallam and Wignall, 1997; Rampino *et al.*, 1997; Toon *et al.*, 1997; Rampino, 1999; Pálfy, 2004; Reimold *et al.*, 2005, 2008; Kelley, 2007; Racki, 2012; and see also Section 4.1 on impact periodicity). The concept of impact-driven mass extinctions led to the concept of an impact kill curve (Raup, 1990, 1992) that correlates extinction magnitude or species exterminated with impact crater size. Chicxulub, it was postulated, was particularly devastating because of its large size. That then begged the question: What was the threshold of an extinction level event? It was subsequently recognized that there may be a family of kill curves that reflect extant ambient and biological conditions at the time of impact (Kring, 2002). Yet, the question remained: What is the threshold size of event or events needed to cause extinction? The community has probed that question in two ways. First, an effort has been made to locate evidence of shock metamorphism at mass extinction horizons, which has generated contradictory results (*e.g*., Retallack *et al.*, 1998 for the end-Permian; and Bice *et al.*, 1992; Patzer *et al.*, 2004; Kring *et al.*, 2017a for the Late Triassic). The second approach has been to locate ejecta from other large impact events and determine if they are correlated with extinctions (*e.g*., Grey *et al.*, 2003; Pálfy, 2004; Clutson *et al.*, 2018).

The Late Devonian Frasnian/Famennian transition, associated with an extinction event, has an age (∼372 Ma) (Percival *et al.*, 2018; cf. Kaufmann, 2006) that is similar to a previously published age of 377 ± 2 Ma for the ≥52 km-diameter Siljan impact structure in Sweden, Europe's largest impact structure (Reimold *et al.*, 2005). However, current Ar–Ar results suggest that the Siljan impact occurred at either ∼400 or ∼380 Ma (Jourdan and Reimold, 2012). Therefore, a causal link with the Frasnian/Famennian boundary event appears implausible (Racki, 2012). Likewise, there is currently no convincing evidence of global-scale impacts at the end-Permian at ∼252 Ma (*e.g*., Retallack *et al.*, 1998; Reimold and Koeberl, 2000; Renne *et al.*, 2004; Wignall *et al.*, 2004), which marks the biggest of all life crises on Earth during which more than 95% of marine species and 70% of terrestrial vertebrates went extinct (*e.g*., Erwin *et al.*, 2002). The event that created the Permo-Triassic ∼40 km-diameter Araguainha impact structure in Brazil, South America's largest impact structure with a U–Pb age of 254.7 ± 2.5 Ma (Tohver *et al.*, 2012), may have had continent-scale effects (Tohver *et al.*, 2013, 2018), but was likely too small to cause a global biological trauma (*e.g*., Walkden and Parker, 2008). A more recent set of geochronologic results, moreover, suggests that the Araguainha impact may be somewhat older (259 ± 5 Ma) (Erickson *et al.*, 2017). Instead, the end-Permian extinction event may have been caused by volcanic activity in large igneous provinces, such as the Emeishan and Siberian Traps in the final stages of the Permian (*e.g*., Shen *et al.*, 2011; Burgess *et al.*, 2017; Ernst and Youbi, 2017) and potentially other compounding environmental factors.

It appears, however, that there may be a small, but measurable, extinction event that is correlated with the Manicouagan impact event around ∼215 Ma (Onoue *et al.*, 2016), which would have produced regional to global environmental consequences (Durda and Kring, 2004; Kring, 2017a) and may be linked to a positive platinum group element anomaly in Upper Triassic deep-sea sediments (Sato *et al.*, 2013). The door on those events has just opened; many more details should be forthcoming now that relevant outcrops have been located for more detailed study. Evidence for impact coinciding with the end-Triassic at ∼201 Ma is somewhat dubious (*e.g*., Olsen *et al.*, 2002; Simms, 2003, 2007; Tanner *et al.*, 2004; Hesselbo *et al.*, 2007; Kring *et al.*, 2007; Schmieder *et al.*, 2010b; Smith, 2011; Lindström *et al.*, 2015), although earlier reports of putative shocked quartz grains at the Triassic/Jurassic boundary in Austria (Badjukov *et al.*, 1987) and Italy (Bice *et al.*, 1992) and an iridium anomaly (Olsen *et al.*, 2002) certainly leave room for new research. The Latest Triassic (Rhaetian) ∼40 km-diameter Rochechouart impact structure in France previously had an age that overlapped with the Triassic/Jurassic boundary (Schmieder *et al.*, 2010a), but new Ar–Ar results suggest that the impact occurred some ∼5 Myr before the transition (Cohen *et al.*, 2017). Similar to widespread volcanism during the end-Permian, the Central Atlantic Magmatic Province (CAMP) may be a driving force of extensive seismicity, emission of gases, and extinction at the end of the Triassic (*e.g*., Marzoli *et al.*, 1999; Lindström *et al.*, 2015; Davies *et al.*, 2017).

Thus far, the only convincing case for impact as the trigger of a mass extinction and severe, global-scale paleoenvironmental effects remains the giant Chicxulub impact on the Yucatán Peninsula in Mexico, which has been stratigraphically, (micro-)paleontologically, geochemically, and in terms of precise U–Pb and Ar–Ar ages linked with the Cretaceous/Paleogene boundary at ∼66.05 Ma (*e.g*., Hildebrand *et al.*, 1991; Kring and Boynton, 1991; Toon *et al.*, 1997; Smit, 1999; Kring, 2007; Schulte *et al.*, 2010; Renne *et al.*, 2013, 2018; DePalma *et al.*, 2019). Some of the hazardous paleoenvironmental effects caused by the Chicxulub impact (see Kring, 2007 for a summary) include a roughly Richter magnitude 10.5 earthquake that, in turn, triggered a large-scale tsunami and, in paleolakes and lagoons, forceful seiches (*e.g*., Smit and Romein, 1985; Bourgeois *et al.* 1988; DePalma *et al.*, 2019); the global distribution of airborne distal impact ejecta (*e.g*., Smit, 1999; Claeys *et al.*, 2002); shock-heating of the atmosphere and widespread wildfires caused by the fallout of hot ejecta (*e.g*., Wolbach *et al.*, 1985; Melosh *et al.*, 1990; Kring and Durda, 2002; Durda and Kring, 2004; Robertson *et al.*, 2013; Belcher *et al.*, 2015); an almost instantaneous phase of “impact winter” caused by atmospheric dust blocking the sunlight (*e.g*., Vellekoop *et al.*, 2014, 2016; Brugger *et al.*, 2017), followed by a superimposed, slower greenhouse effect in response to the voluminous release of atmospherically active gases (*e.g*., water vapor, CO_2_, and SO_x_) from the carbonate- and sulfate-dominated target rock (Kring *et al.*, 1996; Pope *et al.*, 1997; Pierazzo *et al.*, 1998; Kring, 2007); and the acidification of ocean water and leaching of soil due to acid rain (*e.g*., Prinn and Fegley, 1987; Retallack *et al.*, 1987; D'Hondt *et al.*, 1994; Retallack, 1996). At the time of impact, the contemporaneous Deccan trap volcanism in India had already been active (Renne *et al.*, 2015; Richards *et al.*, 2015).

It is worth noting that large impacts, capable of causing widespread havoc and mass extinctions, do not only have detrimental effects on the biosphere. While the end-Ordovician extinction (∼443 Ma) was most likely related to climatic effects and glaciation (*e.g*., Wang *et al.*, 2019), some researchers have argued that frequent impacts during the mid-Ordovician (∼470 to 458 Ma) may have, in fact, boosted biodiversification (Schmitz *et al.*, 2008). A similar biodiversification effect among fossil plankton was also proposed for the Acraman impact in the Ediacaran (Grey *et al.*, 2003), a time when more highly organized organisms emerged (*e.g*., Knoll *et al.*, 2006); stratigraphic and isotopic age constraints for the Acraman impact are, however, relatively imprecise (Schmieder *et al.*, 2015b). Recently, Erickson *et al.* (2019a, 2019b) suggested the ∼2.23 Ga Yarrabubba impact in Western Australia, which potentially affected a Paleoproterozoic “Snowball Earth,” may have been a trigger mechanism for the release of large amounts of water vapor into the atmosphere (Kring, 2003), thereby creating a warming effect that may have helped Earth escape its icehouse state (see also Koeberl *et al.*, 2007b; Koeberl and Ivanov, 2019).

### 4.5. High-precision impact geochronology and its relevance to exo- and astrobiology

Could life have first flourished on Earth beneath the floor of an impact crater? This question (the Impact–Origin of Life Hypothesis) (Kring, 2000, 2019) has not been answered quite yet, but an integral part of it—a temporal component studied in detail using high-precision geochronologic techniques—is a core aspect of this work. As formulated in previous studies suggesting that the origin of life may lie in impact crater settings (*e.g*., Kring 2000, 2003, 2019; Cockell and Lee, 2002; Ryder, 2002; Osinski, 2003, 2011; Cockell, 2006), cooling impact craters that hosted hydrothermal systems are thought to have served as a habitat for microbial life on the early Earth and, possibly, Mars (*e.g*., Abramov and Mojzsis, 2009; Osinski *et al.*, 2013, 2017; Rummel *et al.*, 2014; Grimm and Marchi, 2018; Bowling and Marchi, 2018).

A number of geo-biological paleoenvironmental settings have been proposed as potential loci for the origin and evolution of microbial life on the Hadean–Eoarchean Earth more than 3.8 Ga ago (*e.g*., Nisbet and Sleep, 2001); a recent review is provided by Westall *et al.* (2018). These settings include, among others, sulfide-rich hydrothermal vents (*e.g*., Baross and Hoffman, 1985; Russell and Hall, 1997; Russell and Arndt, 2005; Martin *et al.*, 2008) and hydrothermal-sedimentary crustal settings, in which prebiotic molecules may have been initially produced, stabilized, and complexified as a starting material for organic life (*e.g*., Westall *et al.*, 2018). Impact craters and basins on the early Earth, hosting extensive postimpact hydrothermal systems, would have provided a very similar promising setting (*e.g*., Abramov and Kring, 2004). The largest asteroid impacts on the Hadean and Eoarchean Earth more than 3.7 Ga ago would have created at least ∼40 basins ∼1000 km in diameter and several of order 5000 km-diameter (Grieve, 1980; Kring and Cohen, 2002; Kring, 2003; Grieve *et al.*, 2006) and would have, at the same time, delivered prebiotically relevant elements, such as structurally bound water, carbon, nitrogen, phosphorous, and sulfur (*e.g*., Kring and Cohen, 2002; Pasek and Lauretta, 2008; Svetsov and Shuvalov, 2015; Barnes *et al.*, 2016) (compare Section 4.2 and Fig. 6). However, smaller impact craters some tens of km across would have been much more abundant and saturated Earth's surface (*e.g*., Abramov and Mojzsis, 2009). While the largest of those impact events likely vaporized surface water (Sleep *et al.*, 1989; Zahnle and Sleep, 2006) and produced large amounts of impact melt (*e.g*., Grieve and Cintala, 1992; Grieve *et al.*, 2006), making surface conditions untenable for life, numerical models suggest the subsurface was still habitable (Abramov and Kring, 2004, 2005, 2007; Abramov and Mojzsis, 2009; Grimm and Marchi, 2018). Basin-sized and smaller impacts would have produced subsurface hydrothermal systems conducive for prebiotic chemical reactions that could have led to the early evolution of microbes (*e.g*., Kring, 2000, 2003; Ryder, 2002; Bowling and Marchi, 2018). The volumes of impact-generated habitable zones for mesophilic, thermophilic, and hyperthermophilic microbial life forms in the subsurface of the Hadean–Eoarchean Earth (*i.e*., inside impact craters and the fractured crust below) were significant (of order ∼10^9^ km^3^) (Abramov and Mojzsis, 2009). As with the flux of impactor mass over time (see Section 4.2), the largest impact structures would have provided the most voluminous hydrothermally altered and habitable zones. The volume of rock that sustained habitable temperatures (≤110°C) over hundreds of thousands of years attained up to ∼40,000 km^3^ in larger impact structures ∼200 km across (Abramov and Kring, 2004). The colonization of the central domains of such impact craters may have occurred some ∼20,000 years after the impact (Abramov and Kring, 2004; Abramov and Mojzsis, 2009). This estimate is consistent with the relatively rapid recovery of life at ground zero inside the Chicxulub crater after ∼30,000 years (Lowery *et al.*, 2018).

**FIG. 8.**
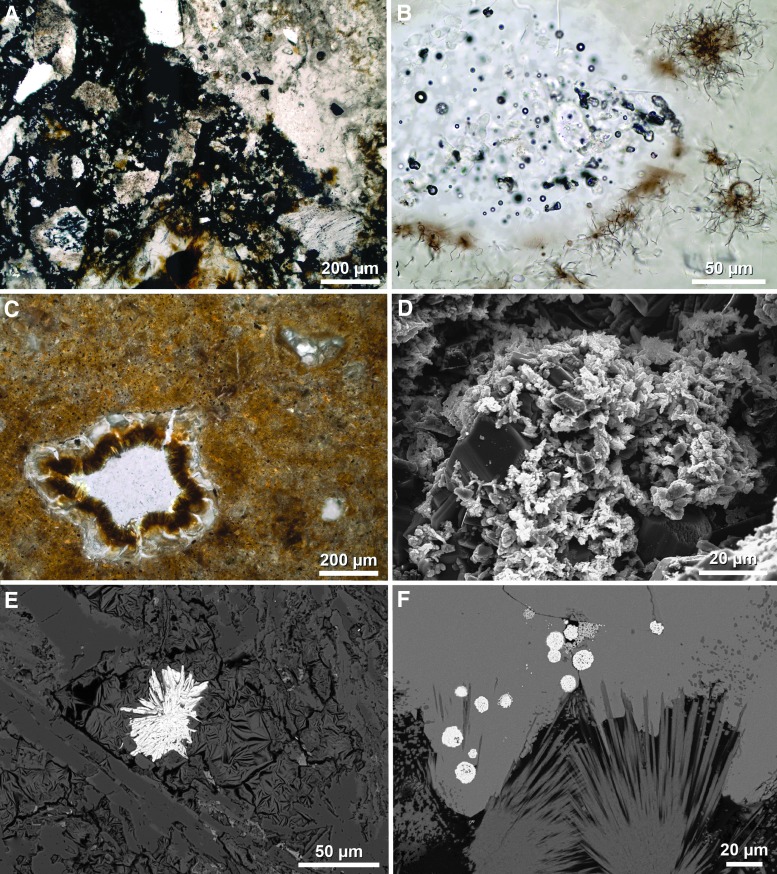
Impact lithologies with biologically relevant elements and/or evidence of hydrothermal alteration as potential analogues for impact crater-hosted microbial habitats. **(A)** Impact melt breccia rich in carbon (enriched in dark interstitial material) from the ∼5 km-diameter Gardnos impact structure, Norway. **(B)** Impact glass from the Nördlinger Ries, Germany, with vesicular domain of silica glass (lechatelierite) and whiskers (trichites) of pyroxene; this type of glass has been linked with possible evidence of fossil microbial life (*e.g*., Lindgren *et al.*, 2010; Sapers *et al.*, 2014, 2015). **(C)** Hydrothermally altered impact melt rock with larger vesicle lined by secondary clay minerals from the ∼80 km Puchezh-Katunki impact structure, Russia. **(A–C)** Optical images, plane-polarized light. **(D)** Altered and locally corroded K-feldspar overgrown by clay minerals in shock-recrystallized and hydrothermally altered granite from the Lappajärvi impact structure, Finland. Unaltered K-feldspar (darker gray) from this sample was used for high-precision Ar–Ar geochronology (Schmieder and Jourdan, 2013a). Secondary electron image. **(E)** Clay alteration domain (gray, with irregular cracks) and secondary barite (Ba-sulfate) in altered impact melt rock from the ∼90 km-diameter Acraman impact structure, South Australia (Williams, 1994; Schmieder *et al.*, 2015b). **(F)** Small pyrite (Fe-sulfide) framboids in zeolite (light gray: analcime; darker gray: Na-dachiardite) in hydrothermally altered reworked suevitic breccia from the 180 km-diameter Chicxulub impact crater (Kring *et al.*, 2017b). **(E, F)** Backscattered electron images.

Although large impacts were much more abundant during the Hadean and Archean before ca. 3.7 Ga (*e.g*., Turner *et al.*, 1973; Tera *et al.*, 1974; Ryder, 1990; Kring and Cohen, 2002; Bottke and Norman, 2017), impact craters and their hydrothermally altered rocks and minerals accessible on Earth today (*e.g*., Allen *et al.*, 1982; Osinski *et al.*, 2001, 2013; Zürcher and Kring, 2004; Naumov, 2005; Kring *et al.*, 2017b) are valuable analog sites for the type of impact-produced, wet, and warm habitat described above. Putative fossils of microbial life found in hydrothermally altered impact glass, for example, at the early Cretaceous, 19 km-diameter Dellen impact structure in Sweden (Lindgren *et al.*, 2010) and the Miocene Ries crater in Germany (Sapers *et al.*, 2014, 2015), as well as sulfur isotopic signatures indicating microbial reduction of target rock sulfate at the Miocene, ∼24 km-diameter Haughton impact structure, Canada (Parnell *et al.*, 2010), and the latest Triassic, ∼40 km-diameter Rochechouart impact structure, France (Simpson *et al.*, 2017), may be evidence for the colonization of impact crater-hosted habitabile zones by thermophilic microbes. Figure 8 shows a variety of impactites typically found in terrestrial impact structures, including lithologies enriched in biologically relevant elements (such as carbon and sulfur) and hydrothermally altered rocks that may represent analogues for the setting in impact crater-hosted microbial habitats (*e.g*., Kring, 2000, 2003; Ryder, 2002; Cockell *et al.*, 2003; Cockell, 2006).

**FIG. 9.**
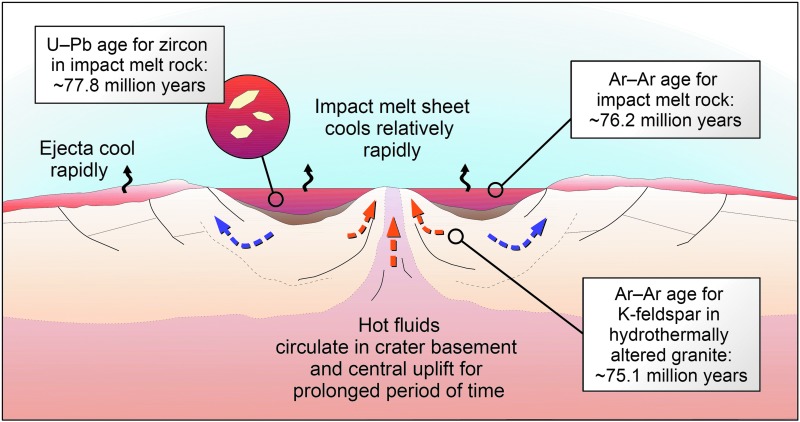
Schematic illustration of a cooling complex impact crater (cross-sectional view), for example, the ∼23 km-diameter Lappajärvi impact structure in Finland, modified after Schmieder and Jourdan (2013b). High-precision geothermochronologic results for different types of lithologies can resolve the crater cooling process. Whereas the impact melt sheet and impact eject cool relatively fast, the central uplift of the structure maintains the circulation of hot fluids for a prolonged period of time. The hottest temperature in that hydrothermal system occurs in the central, uplifted domain of the impact crater; whereas fluids in the crater rim domain are comparatively cool (compare Abramov and Kring, 2004, 2005, 2007). Age values indicated are actual results for Lappajärvi, taken from Schmieder and Jourdan (2013a) and Kenny *et al.* (2019b). Uranium–lead and Ar–Ar results for rapidly cooled impact ejecta (*e.g*., ejected shocked zircon grains and tektites) have, thus far, provided the best-estimate age for impact events. In contrast, hydrothermally altered rocks and minerals commonly yield ages reflecting protracted postimpact fluid flow that can locally last for >1 Myr in impact structures >20 km in diameter (*e.g*., Schmieder *et al.*, 2018a; Kenny *et al.*, 2019b).

In addition to habitable volumes and substrates, two key factors in hot fluid systems as biological habitats are their temperature and lifetime. Geochronologic studies and numerical modeling suggest that the largest terrestrial impact craters, such as Sudbury and Chicxulub, may have sustained initially hot (>300°C) hydrothermal systems for more than 2 Myr (*e.g*., Ames *et al.*, 1998; Abramov and Kring, 2004, 2007; Zürcher and Kring, 2004), whereas medium-sized impact craters around 20–30 km in diameter were generally thought to cool down more rapidly, perhaps over a few thousands or tens of thousands of years (*e.g*., Pohl *et al.*, 1977; Osinski *et al.*, 2001). Recent high-precision U–Pb and Ar–Ar results for the 23 km-diameter Lappajärvi impact crater in Finland, however, suggest those initial estimates may have been too conservative. An older zircon U–Pb age of ∼77.85 Ma, recording lead diffusion at ∼900°C (Kenny *et al.*, 2019b), in combination with significantly younger Ar–Ar results of ∼76 to 75 Ma for impact melt rock and K-feldspar that record argon diffusion at ∼400 to 200°C over several hundred thousand years (Schmieder and Jourdan, 2013a), indicates that even the comparatively small Lappajärvi crater cooled down from initially hot, impact melt-producing temperatures (>2000°C) (Bischoff and Stöffler, 1984) to hotter-than-habitable conditions over a period of at least 1.3 Myr (Kenny *et al.*, 2019b). This demonstrates that modern isotopic techniques have the capacity to resolve various stages of an impact event as a protracted geologic process rather than an instantaneous event. It is becoming more apparent that the most precise and accurate impact ages are obtained by using high-temperature geochronometers and/or, if available, rapidly cooled (distal) impact melt lithologies that landed (far) outside their hot and slowly cooling source crater (Schmieder *et al.*, 2018a; Kenny *et al.*, 2019b). More importantly, the slow cooling of the Lappajärvi crater resolved by combined high-resolution U–Pb and Ar–Ar geochronology makes medium-sized impact craters (∼20 to 30 km in diameter), which are orders of magnitude more common over geologic time than Sudbury- or Chicxulub-sized craters (*e.g*., French, 1998), an important type of habitat for thermophilic and hyperthermophilic microbes on the early Earth (Kring, 2000, 2003; Cockell *et al.*, 2003; Cockell, 2006). A scheme of a slow-cooling complex impact crater, such as Lappajärvi, is shown in Fig. 9. Slowly cooling impact crater-hosted hydrothermal systems similar in volume and lifetime to that at Lappajärvi are, therefore, also relevant to astro- and exobiology. In analogy to Earth, medium-sized impact craters on early (Noachian) Mars may have been an important extraterrestrial habitat, as well (*e.g*., Newsom, 1980; Newsom *et al.*, 1986, 2001; Rathbun and Squyres, 2002; Abramov and Kring, 2005; Schwenzer and Kring, 2009; Osinski *et al.*, 2013; Rummel *et al.*, 2014; Abramov and Mojzsis, 2016).

## 5. Conclusions

This work presents a comprehensive collection of revised ages for terrestrial impact structures and deposits. Impact geochronology and the use of the U–Pb and Ar–Ar techniques and other methods have significantly refined the time line for a number of impact events on Earth, whose ages can be correlated with other impacts and geologic events in Earth history, and which can be used to assess the impact (mass) flux on Earth through geologic time. Based on the latest geochronologic results, synchronous double impacts on Earth seem to be rare, and evidence for a large-scale multiple impact event on our planet is currently missing. However, the Ordovician marks a time period of intense bombardment over several million years, supported by a growing number of Ordovician U–Pb, Ar–Ar, and stratigraphic impact ages. Only the Chicxulub impact at the K/T boundary 66.05 Myr ago has been firmly linked to a mass extinction event, in part, based on high-precision U–Pb and Ar–Ar results. The latter can also be used to determine the lifetime of hydrothermal systems in cooling impact craters, as recently done for the slowly cooled Lappajärvi impact crater in Finland as an analog site for impact crater-hosted habitats for microbial life on the early Earth and, possibly, Mars.

## Notes

Associate Editor: Christopher McKay

## Abbreviations Used

CA-TIMS: chemical abrasion thermal ionization mass spectrometry
ICS: International Chronostratigraphic Chart
LA-ICP-MS: laser ablation inductively coupled plasma mass spectrometry
MSWD: mean square weighted deviation
SHRIMP: sensitive high-resolution ion microprobe
SIMS: secondary ion mass spectrometry
